# Determinism of microbial community assembly by drastic environmental change

**DOI:** 10.1371/journal.pone.0260591

**Published:** 2021-12-02

**Authors:** Akifumi Nishida, Mayuko Nakagawa, Masayuki Yamamura

**Affiliations:** 1 Department of Electrical Engineering and Bioscience, Waseda University, Tokyo, Japan; 2 School of Computing, Tokyo Institute of Technology, Ookayama, Kanagawa, Japan; 3 Earth-Life Science Institute, Tokyo Institute of Technology, Tokyo, Japan; University of Hong Kong, HONG KONG

## Abstract

Microbial community assembly is shaped by deterministic and stochastic processes, but the relationship between these processes and the environment is not understood. Here we describe a rule for the determinism and stochasticity of microbial community assembly affected by the environment using *in silico*, *in situ*, and *ex situ* experiments. The *in silico* experiment with a simple mathematical model showed that the existence of essential symbiotic microorganisms caused stochastic microbial community assembly, unless the community was exposed to a non-adapted nutritional concentration. Then, a deterministic assembly occurred due to the low number of microorganisms adapted to the environment. In the *in situ* experiment in the middle of a river, the microbial community composition was relatively deterministic after the drastic environmental change caused by the treated wastewater contamination, as analyzed by 16S rRNA gene sequencing. Furthermore, by culturing microbial communities collected from the upstream natural area and downstream urban area of the river in test tubes with varying carbon source concentrations, the upstream community assembly became deterministic with high carbon concentrations while the downstream community assembly became deterministic with low carbon concentrations. These results suggest that large environmental changes, which are different from the original environment, result in a deterministic microbial community assembly.

## Introduction

Microbial communities in the human gut, floral nectars, and bioreactors are shaped by not only deterministic but also stochastic processes, which result in different microbial communities even in the same environment [1]. The deterministic convergence and stochastic variation among microbial community replicates have been studied with various systems, such as bottom-up synthetic microbial communities with a few microbial species and microbial communities cultivated from the natural environment. Previous research has used the bottom-up synthetic microbial communities to study population density fluctuations around replicate-average dynamics in three microbial species communities [2], stochastic assembly in the host by two bacterial species [3], priority effects among four yeast species [4], and high-order interactions among three bacteria [5]. These results are important to know the core rules of microbial community assembly; however, it is also necessary to extract the rules for stochastic processes from communities consisting of many microorganisms. This can be done by cultivating microbial communities collected from *in situ* environments, and analyzing 16S rRNA gene sequences [6, 7]. Interestingly, assembled microbial composition in communities from the surface of leaves were taxonomically diverse at the genus level, but converged at the family level [7]. This suggests that many genus-level taxa have similar functions to adapt to the environment, but family-level taxa have different functions to adapt. However, the impact of the environment on the stochasticity of microbial community assembly remains unknown.

**Fig 1:**
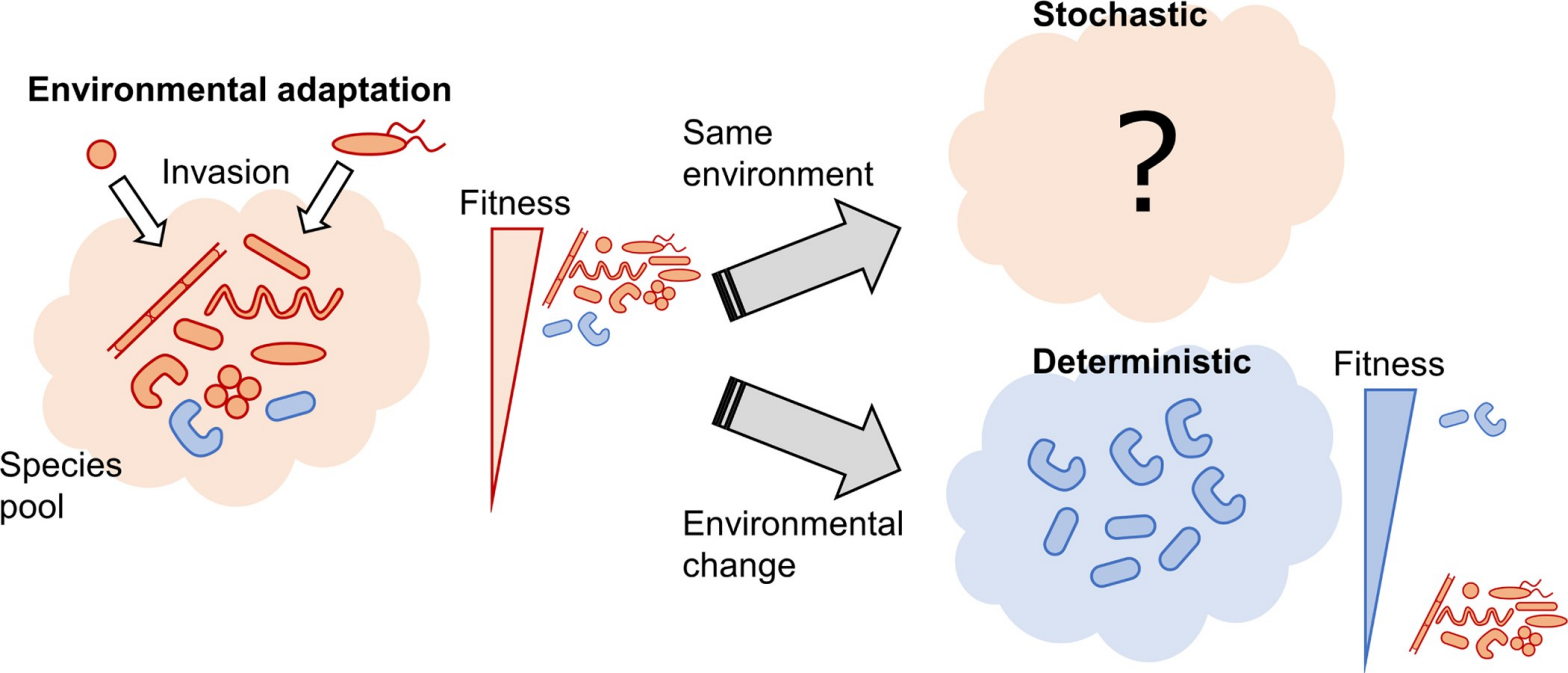
Schematic of a rule proposing deterministic assembly by environmental change. If the environment changes from its original state that fits many pooled microorganisms, the assembly of microbial communities may become deterministic due to the limitation of possible self-organization patterns.

In this study, to determine a rule for the determinism and stochasticity of microbial community assembly, the hypothesis that microbial community assembly becomes deterministic by large environmental change was examined in *in silico*, *in situ*, and *ex situ* experiments (Fig 1). In the *in silico* experiment, by constructing a mathematical model in which the traits of nutrient intake differed among microbial species and adding an effect of essential microorganisms, the stochasticity of the microbial community assembly was examined by adding noise to initial microbial abundances. Then, by virtually constructing microbial communities adapted to high/low nutritional concentrations, which consisted of many microorganisms adapted to high/low nutritional concentrations, the stochasticity of the microbial community assembly was examined when exposed to non-adapted nutritional concentrations. In the Tama River *in situ* experiment, we examined the stochasticity of the microbial community composition where the river environment changed drastically after mixing with the treated wastewater. Furthermore, by culturing microbial communities collected upstream and downstream of the Tama River in test tubes with varying carbon source concentrations, the effect of the combination of the original environment and the culturing environment on the stochasticity of microbial community assembly was examined.

## Materials and methods

### Ethics statement

We sampled water from the Tama river in public space for this study. The Ministry of the Environment in Japan does not restrict to sample water (~10 L) from the Tama River in public space.

### Mathematical model of the microbial community

**Fig 2:**
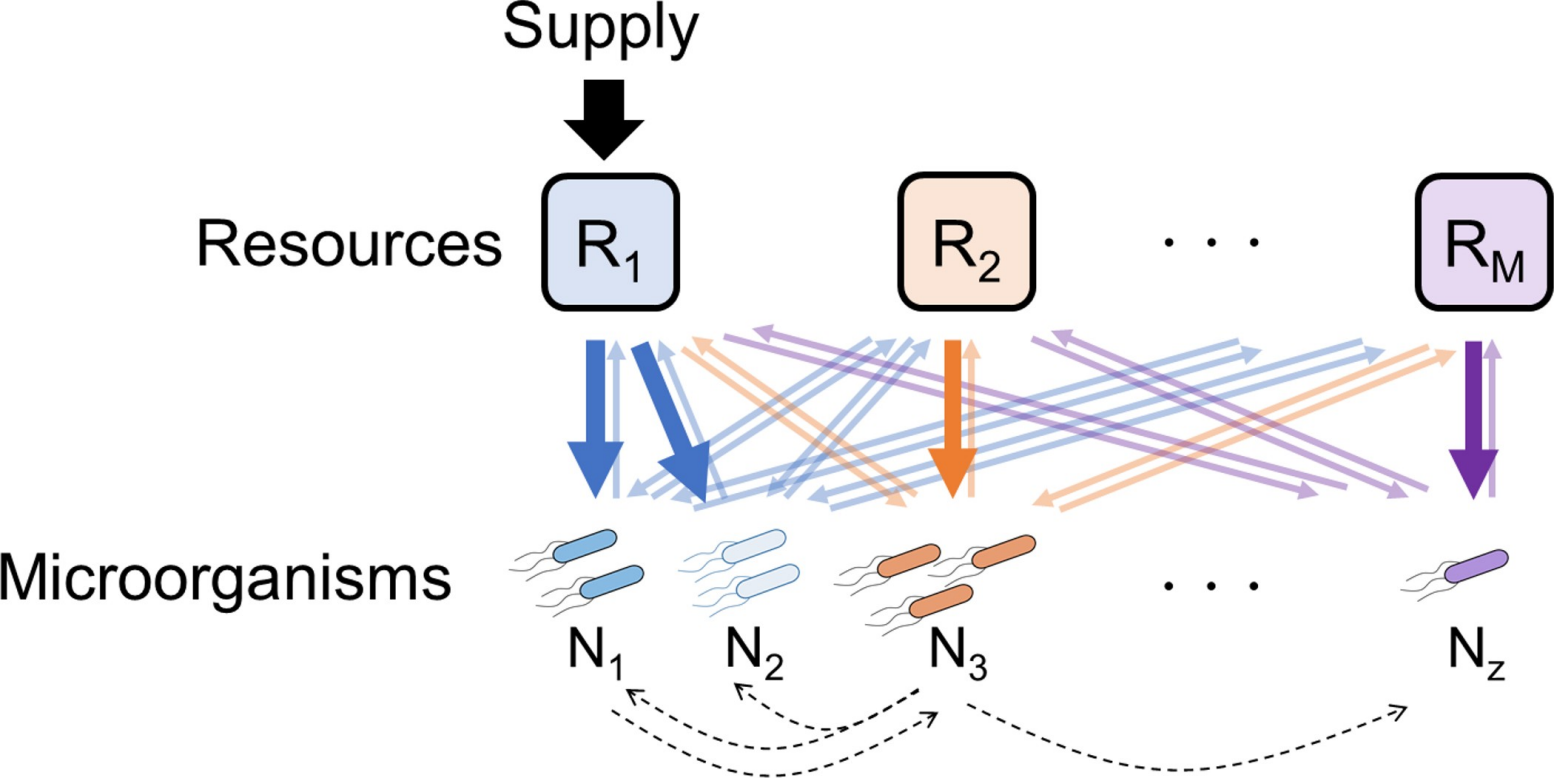
*In silico* model design. Each microbial species uptakes resources and secretes metabolic byproducts. Arrows from resources to microbial species indicate resource uptake and opposite arrows indicate secretion of metabolic byproducts. All resources are associated with specialized microbial species, as indicated by thick arrows. Each microbial species has essential symbiotic microorganisms, indicated by dashed arrows. For example, the metabolism of the microbial species *N*_*1*_ is activated by the existence of microbial species *N*_*3*_.

The mathematical model used in this study was based on MacArthur’s consumer resource model modified by J. E. Goldford et al. (Fig 2) [7]. The model was further modified by the addition of the effect of essential symbiotic microorganisms and the intake specialty for a specific resource concentration. The modified mathematical model was as follows:

$$\frac{{dN}_{i}}{dt}=\sum_{\alpha }F_{i}G_{i\alpha }C_{i\alpha }R_{\alpha }N_{i}-m_{i}N_{i}$$ (1)

$$\frac{{dR}_{\alpha }}{dt}=H_{\alpha }-\sum_{i}F_{i}G_{i\alpha }C_{i\alpha }R_{\alpha }N_{i}+\sum_{\beta ,i}F_{i}G_{i\alpha }D_{\alpha \beta }^{i}C_{i\alpha }R_{\alpha }N_{i}$$ (2)

where *N*_i_ and *R*_α_ represent the biomass of microbial species *i* and the amount of resource *α* (S1 Table). In Eq (1), the first term indicates microbial growth by consuming resources and the second term indicates the death and dormancy of microorganisms. Resource consuming rates are decided by effects of essential symbiotic microorganisms *F*_*i*_, specialization for a specific resource concentration *G*_*iα*_, and a species-specific resource *α* consuming rate *C*_*iα*_. $F_{i}=\frac{s_{i}}{K_{i}+s_{i}}$, in which *s*_*i*_ represents the total amount of essential microorganisms for species *i* and *K*_*i*_ represents a value similar to the Michaelis constant, indicates that a decrease in the total essential microorganism amount *s*_*i*_ represses the resource consuming activity of species *i*. $G_{i\alpha }=4\frac{I_{\alpha }}{L_{i}+I_{\alpha }}\frac{L_{i}}{L_{i}+I_{\alpha }}$, in which *I*_*α*_ represents the supply concentration of resource *α* into the medium and *L*_*i*_ represents a value similar to the Michaelis constant, indicates that the specific resource concentration activates the resource consuming activity of species *i* (S1 and S2 Figs). In Eq (2), the first term indicates the resource supply, the second term indicates resource consumption, and the third term indicates the secretion of metabolic byproducts. For simplicity, it was assumed that only one resource was supplied to the communities and other resources were produced by metabolic activity. $H_{\alpha }=\frac{I_{\alpha }-R_{\alpha }}{\tau_{\alpha }}$, in which *τ*_*α*_ represents the transfer rate, denotes the supply resource. *D*^*i*^_*αβ*_ depicts the stoichiometric matrix from resource *β* to *α*. Microorganisms in the model can be considered groups of microbial species, defined as a “*guild*” in ecology [8], with each group exploiting resources and interacting with other groups in a similar way.

### Numerical calculation of microbial community assembly

For numerical calculation using the above mathematical model, the Monte Carlo method was used. As a testbed, it was assumed that one of 10 resources was continuously supplied, and the resources were uptaken and secreted as metabolic byproducts by microorganisms. The species-specific resource *α* consuming rate *C*_*iα*_ was decided with a rule that each species specialized for a resource *f*_*i*_ as in J. E. Goldford et al. [7]. We first sampled *C*_*i*,*α = f*_ as: $C_{i,{\alpha =f}_{i}}∼Normal(\mu ,\sigma^{2})$, where Normal(*μ*, *σ*^2^) represents a normal distribution of mean *μ* and standard deviation *σ*. *C*_*i*,*α = fi*_ was set between 0–1. Next, we sampled *C’*_*i*,*α≠fi*_ by uniform distribution, *C’*_*i*,*α≠fi*_ ~Uniform(0, 1). *C*_*i*,*α≠fi*_ was set as $C_{i,\alpha \neq f_{i}}=(1-C_{i,\alpha =f_{i}})\frac{C_{i,\alpha \neq f_{i}}^{\prime }}{\sum_{\gamma \neq f_{i}}C_{i,\gamma }^{\prime }}$. For all calculations, we selected *μ* = 0.4 and *σ* = 0.01.

To examine changes in the supplying resource concentration, two communities were assumed; each adapted to a high or low resource concentration, respectively (S1 Fig). We chose the adapted resource concentration as *L*_*i*_ = 100 for the high concentration and *L*_*i*_ = 1 for the low concentration (S2 Fig). For simplicity, it was assumed that only species group *S* specialized for a supplied resource *f*_*s*_ had $G_{i\in S,\alpha =f_{s}}=\frac{I_{\alpha }}{L_{i}+I_{\alpha }}\frac{L_{i}}{L_{i}+I_{\alpha }}$ for the supplied resource and that for other resources or other species, $G_{i\in S,\alpha \neq f_{s}}=1$ and *G*_*i*∉*S*,*α*_ = 1. The community adapted to a high resource concentration consisted of 45 and five species adapted to a high and low resource concentration, respectively, for a supplied resource, and 10 species specialized for each of the other resources. The effect of essential-symbionts $F_{i}(s_{i})=\frac{s_{i}}{K_{i}+s_{i}}$ was required for stochasticity. The number of essential-symbionts for each microorganism was 10, which were randomly selected among species specialized for other resources. As a supply resource concentration *I*_*α*_, *I*_*α*_ = 100 was examined as supply of a high resource concentration and *I*_*α*_ = 1 as supply of a low resource concentration (S2 Fig for other concentrations). Other parameters were *K*_*i*_ = 0.01, *m*_*i*_ = 0.05, and τ_α_ = 1. Each numerical calculation was performed 10 times by changing the initial biomass, which were randomly selected between 0.01 and 1. Renkonen similarity was calculated for each of the 10 replicates using the final microbial compositions. The repeated calculation was further assessed 100 times by rechoosing the resource consuming rate *C*_*iα*_, stoichiometric matrix *D*^*i*^_*αβ*_, and essential symbionts (S3 Fig).

To examine changes in the number of species comprising the community, the model was the same as the abovementioned for the examination of changes in the supplying resource concentration, but the adaptation to a specific resource concentration was not added, with *G*_*i*,*α*_ = 1 in Eqs (1) and (2). When the number of species comprising the community changed, each number of species specialized for one of 10 resources equalized. Source codes for these numerical calculations are available at https://github.com/Nishida12/Stochastic_community_assembly.

### Sample site and sample collection

The Tama River is a major flowing border between the Tokyo and Kanagawa prefectures. The river spans 138 km in length and flows through both natural and urban areas. In such a river where the environment changes drastically, river water was collected once every two months at eight sites for a year (S4 Fig). The drastic change occurred by contamination of treated wastewater from two wastewater treatment plants between site 4 to 5 and four plants between site 5 to 6 [9]. The sampling period was from August 31, 2018 to July 21, 2019. The sampling days and the day before were not rainy. A prewashed bucket tied with string was thrown into the river 5 m away from the shore to collect river water. Water (300 mL) was collected into prewashed polypropylene bottles (AS ONE, Osaka, Japan) for DNA isolation and into sterilized centrifuge tubes (20 mL water, AGC, Tokyo, Japan) after the water was filtered using a 0.22 μm filter (Merck, Darmstadt, Germany) for ion chromatography and total organic carbon (TOC) measurements. Water samples (300 mL) were stored on ice and DNA was isolated within 10 h. River temperature, dissolved oxygen, and pH were measured *in situ* using a DO200A for temperature and dissolved oxygen (Xylem, Rye Brook, NY, USA) and a PH-6600 for pH (CUSTOM, Tokyo, Japan) (S2 and S3 Tables).

### DNA isolation, polymerase chain reaction (PCR) amplification, sequencing, and data availability

Genomic DNA in river water was isolated using the DNeasy PowerWater Sterivex DNA Isolation kit (Qiagen, Hilden, Germany). The liquid feed pump was prewashed with 50 mL of river water, and 250 mL was used for DNA isolation. Genomic DNA from *ex situ* cultures was isolated using the NucleoSpin Microbial DNA kit (Macherey-Nagel, Düren, Germany). 16S rRNA gene amounts were quantified by real time-PCR using PowerUp SYBR Green Master Mix (Thermo Fisher Scientific, Waltham, MA, USA) and U16SRT primers [10]. The PCR amplification methods for the 16S rRNA gene V3 and V4 variable regions and MiSeq sequencing (Illumina, San Diego, CA, USA) using the MiSeq Reagent Kit v3 (600 Cycles) (Illumina, San Diego, CA, USA) have been previously described [11]. Sequencing data were deposited to the National Center for Biotechnology Information (Accession: PRJNA649941).

### Taxonomic classification based on 16S rRNA gene sequences, biodiversity analysis, and estimation of the functional gene profile

The QIIME2 docker software (qiime2-2019.10) was used to analyze the 16S rRNA gene sequences [12]. *In situ* and *ex situ* 300 nucleotide (nt) paired-end reads were trimmed using the *qiime dada2 denoise-paired* command (20–296 nt for forward and 20–250 nt for reverse). The reads were taxonomically classified with 99% amplicon sequence variant (ASV) data using SILVA 132 [13] and the *qiime feature-classifier extract-reads* command (CTACGGGGGGCAGCAG for forward and GGACTACCGGGGTATCT for reverse). The ASV tables of *in situ* and *ex situ* experiment are shown in S1 and S2 Datasets, respectively. α-diversity was calculated by QIIME2 with a sampling depth of 10,000 for *in situ* and 180,000 for *ex situ* reads. Metagenome prediction based on 16S rRNA sequence was conducted using PICRUSt2 (2.1.4-b) in QIIME2 [14]. Pathway abundance data from PICRUSt2 were converted to relative abundances and principal coordinate analysis (PCoA) based on Bray-Curtis dissimilarity was conducted using R stats package (version 4.0.3) [15]. The difference of PICRUSt2 data between upstream (site 1–4) and downstream (site 5–8) were tested by permutational multivariate analysis of variance (PERMANOVA) based on Bray-Curtis dissimilarity using R vegan package (version 2.5.7) [16].

### Ion chromatography and dissolved organic carbon (DOC) analysis of river water

Water samples for carbon isotope analysis of DOC were collected using 50 mL syringes and filtered with membrane syringe filters with a pore size of 0.20 μm (DISMIC–25AS; Advantec Toyo Kaisha, Tokyo, Japan). Filtered water (5 mL) was subsampled and 0.2 vol % HCl was added to remove the dissolved inorganic carbon. DOC concentrations of acidified water samples were then measured in triplicate using a TOC analyzer (TOC-5000; Shimadzu, Kyoto, Japan).

Anion concentrations were measured via ion chromatography on a Shimadzu Ion Chromatograph (Shimadzu, Kyoto, Japan) equipped with a Shodex SI-90 4E anion column (Showa Denko, Tokyo, Japan). A Shim-pack IC-C4 (Shimadzu, Kyoto, Japan) was used without a suppressor for cations.

### *Ex situ* cultivation of the bacterial community

Bacterial communities (in 300 mL of water) were sampled from site 2 (upstream) and site 8 (downstream) on September 7, 2019. Water temperature was 22.4°C at site 2 and 27.9°C at site 8. These samples were stored on ice for 5 h, and 150 μL was diluted into 3 mL M9 medium, which was prepared according to the protocol by A. Geerlof [17], while the final glucose concentration as a carbon source was changed to 0.4%, 0.04%, and 0.004%. Each glucose condition had five replicates. Polypropylene cultivation tubes (14 mL, Corning, Corning, NY, USA) were shaken at 180 rpm. According to J. E. Goldford et al. [7], cultures grew 48 h at 25°C, and then 150 μL of each culture was diluted into 3 mL fresh M9 medium. Cultures were passaged 12 times. In the first and second cultures, 200 μg/mL cycloheximide was added to inhibit eukaryotic growth. Cell density was measured immediately before culture dilution using a CO8000 cell density meter (Harvard Bioscience, Holliston, MA, USA) at a 600 nm wavelength. Finally, 2.5 mL of each culture was used for genomic DNA isolation.

### Quantification of stochastic assembly

To quantify the stochasticity of community assembly, Renkonen similarity was used as measure of beta diversity between communities, as previously reported to quantify the stochastic assembly of bacterial communities on leaves at each taxon level [7]. Renkonen similarity is the opposite index but shares the same mechanism as that of Bray-Curtis dissimilarity for comparing relative abundances, as determined by $D(x,y)=1-\frac{1}{2}\sum_{i}\left|x_{i}-y_{i}\right|$, where *x*_*i*_ and *y*_*i*_ are the relative abundances of microbial species *i* in the compared communities. For comparison of *in situ* or *ex situ* communities, genus level taxa were used. Renkonen similarity calculates the similarity between two samples and shows a value between 0–1. Calculating the similarity of all replicate pairs, the stochastic assembly of communities under a condition was quantified. A larger similarity indicated that the community assembly was relatively deterministic, and the converse indicated stochastic assembly.

## Results

### Stochastic assembly occurred by adapted species richness *in silico*

**Fig 3:**
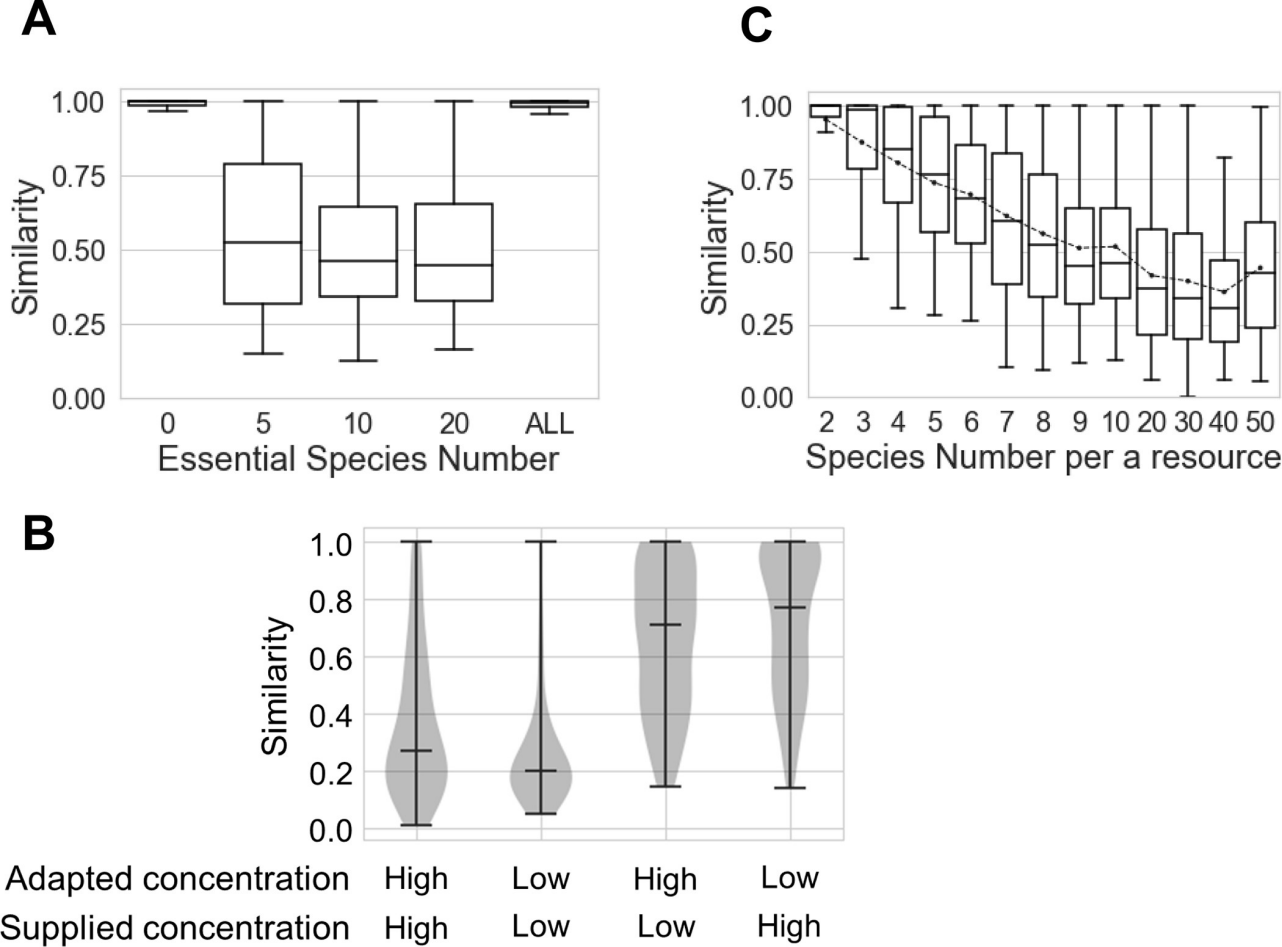
Stochasticity of *in silico* microbial community assembly. **A.** The number of essential-microbial species for each species varied in the community consisting of 10 resources and 100 microbial species, where each resource was specialized in 10 microbial species. **B.** Microbial communities adapted to a high/low concentration resource were supplied with a high/low concentration resource. Horizontal lines in the violin plot represent the maximum, minimum, and median values of similarity. **C.** The number of microbial species specializing in each resource varied. The number of resources is 10; therefore, for example, species number 2 means the community consists of 20 microbial species. Dots in boxes represent the mean of similarity.

To comprehensively understand how stochastic assembly of microbial communities occurred, a numerical calculation with a mathematical model was useful. However, past mathematical models of microbial communities, in which particular ecological traits of microbial species for nutrient intake and interspecies interactions are hypothesized, cannot generate a stochastic assembly even if the initial abundance of each bacterial species is randomly chosen [7]. Therefore, we supposed the existence of essential-microbial species for each microbial species that generated stochasticity of microbial community assembly based on the different initial microbial abundances (Fig 3A). With regard to essential species, each microbial species had some essential species required to live and too little of all essential species was supposed to repress the growth of the species itself (Materials and methods: Mathematical model of microbial community). If all species did not require the existence of any essential species, the community assembly became deterministic (mean of similarity 0.98 among duplicates with varying initial microbial abundances). If all species were essential species for each species, suggesting loss of specific microbial essentiality, the community assembly also became deterministic (similarity 0.98). However, if a certain number of species became essential species, stochastic assembly occurred; namely in this experiment, if the number of essential species for each microbial species was 5, 10, and 20 in 100 species communities, the similarities were 0.55, 0.52, or 0.50, respectively.

As the stochastic assembly caused by the existence of essential species was confirmed, we next prepared microbial communities adapted to high or low resource concentrations *in silico* and examined the effects of changes in environmental resource concentration on stochastic assembly (Fig 3B). The communities adapted to high/low resource concentration consisted of a large number of species adapted to high/low resource concentrations and a small number of species adapted to low/high resource concentrations. If each community adapted to high/low resource concentration was supplied a resource of a corresponding high/low concentration respectively, similarities were 0.37 and 0.26. However, the disagreement between the adaptation concentration and the supplied concentration of a resource suggested drastic environmental change made the community assembly relatively deterministic, with 0.66 and 0.72 of adaptation to high/low concentration (*p* < 0.001, *U* = 2044 for adaptation to high concentration, *U* = 835 for adaptation to low concentration, agreement vs. disagreement, Mann-Whitney *U* two-sided test). One difference of the agreement and disagreement between the adaptation and the supplied concentration was the number of surviving bacterial species; namely, the agreement communities had more surviving microbial species than the disagreement communities. Therefore, to better understand what changed the degree of the stochasticity, we changed the number of community species (Fig 3C). When each of all 10 resources had two microbial species specialized for the resource, i.e. the community consisted of 20 microbial species, the community assembly was closely deterministic (similarity 0.95). Increases in the species number made the communities stochastic, and the stochasticity progressed until the number of species per a resource reached 10, i.e. 100 community species, in our simulation using a simple model (similarity 0.52 ± 0.24). These results suggested that species richness made the community assembly stochastic.

### Environmental change made microbial communities deterministic *in situ*

**Fig 4:**
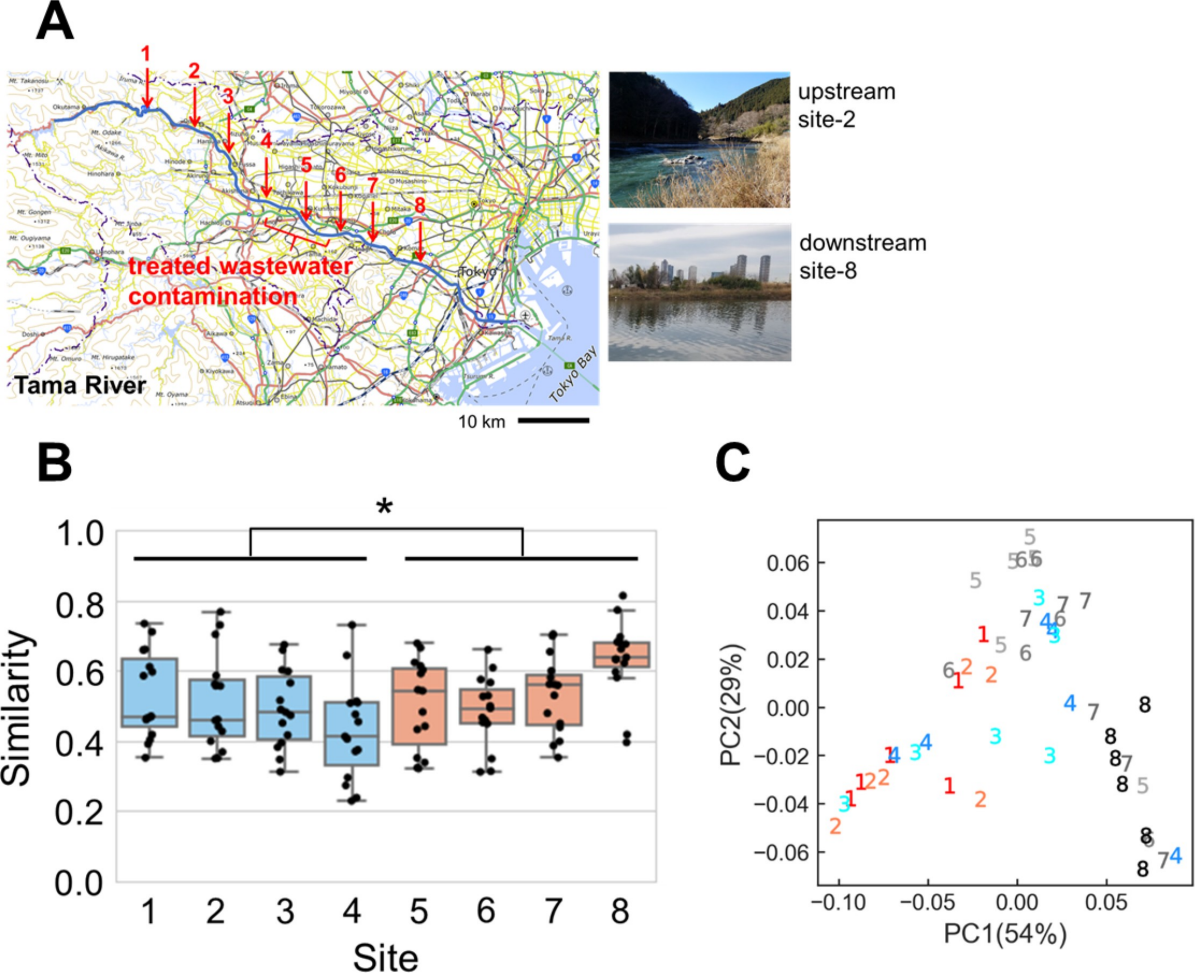
Stochasticity of microbial community assembly in a river. **A.** Microbial communities have been sampled six times at eight sites in the Tama River from natural and urban areas. The map is based on the Digital Map published by Geospatial Information Authority of Japan (2021). **B.** Similarity of bacterial compositions at each site was calculated as assembly stochasticity and compared between before and after contamination of treated wastewater (*p* = 0.028) with box and jitter plot. **C.** Community functional data were analyzed by principal coordinate analysis (PCoA) based on Bray-Curtis dissimilarity. Each number indicates sampling site.

Since drastic environmental change was confirmed in the *in silico* experiment that limited community species richness and made microbial communities deterministic, the rule of stochasticity in nature was examined. For the *in situ* experiment, the Tama River, flowing through both natural and urban areas, was targeted because a drastic change occurred with contamination of treated wastewater from wastewater treatment plants between sites 4 to 6 (Fig 4A). The results of ion chromatography and DOC analysis showed that ionic compounds and DOC increased: especially NO_2_^−^, PO_4_^3−^, and SO_4_^2−^ (S5 and S6 Figs). NO_2_^−^, PO_4_^3−^, and SO_4_^2−^ drastically increased between sites 4 and 5 (NO_2_^−^, 13.2 ± 3.5 standard deviation (SD) to 206.0 ± 106.2 SD [mg/L]; PO_4_^3−^, 5.1 ± 1.6 SD to 27.5 ± 12.0 SD [mg/L]; SO_4_^2−^, 10.8 ± 2.3 SD to 27.7 ± 10.4 SD [mg/L]). DOC increased between sites 3 and 5 (site 3, 2.91 ± 0.43 SD; site 4, 3.46 ± 0.97 SD; site 5, 3.63 ± 0.55 SD), but decreased between sites 7 and 8 (site 7, 3.63 ± 0.72 SD; site 8, 3.10 ± 0.47 SD). Amounts of partial genomic DNA from bacteria also increased in the river downstream (*p* < 0.001, *W* = 17, site 1–4 vs. 5–8, Wilcoxon signed-rank two-sided test; 12.2 ± 19.1 SD vs. 49.2 ± 52.1 SD [pg/μL]) (S7 Fig). These results suggested that treated wastewater contamination made the river eutrophic, and bacteria increased in the downstream environment. It was also possible that DNA contamination from dead microorganisms in sterilized treated wastewater increased after site 4, but observed operational taxonomic units (OTUs) at site 5 (1,783 ± 392 SD) moderately increased compared to that at site 4 (1,292 ± 580 SD) (*p* = 0.17, *U* = 9, Mann-Whitney *U* two-sided test) and observed OTUs at site 8 (993 ± 366 SD) did not differ significantly to those at sites 1–4 (*p* = 0.45, *U* = 87, Mann-Whitney *U* two-sided test) (S8 Fig).

In a river where the environment changed drastically, bacterial composition at six time points was measured (S9 Fig, S2 Table). At the genus level, for example, relative abundances of *Limnohabitans* (site 1, 0.017 ± 0.007 SD; site 8, 0.138 ± 0.070 SD), *Fluviicola* (site 1, 0.007 ± 0.003 SD; site 8, 0.061 ± 0.058 SD), and *Pseudarcicella* (site 1, 0.010 ± 0.008 SD; site 8, 0.053 ± 0.044 SD) were increased along the river (S10 Fig). Using the bacterial composition at six time points and eight places, we calculated similarities among bacterial compositions at each place to quantify the *in situ* stochasticity (Fig 4B). Upstream similarities in sites 1–4 (0.49 ± 0.13 SD) were less than downstream similarities in sites 5–8 (0.54 ± 0.12 SD) (*p* = 0.056, *U* = 1436, site 1–4 vs. 5–8, Mann-Whitney *U* two-sided test), which suggested the assembly of the downstream bacterial community was relatively deterministic compared to that of the upstream community. PCoA biplot based on Bray-Curtis dissimilarity of microbial composition suggested that pH, organic, and inorganic substances were related with the change in microbial composition before and after contamination of treated wastewater (S11 Fig). Community functions predicted by PICRUSt2 showed that the upstream and downstream communities were functionally different, according to the PCoA results based on Bray-Curtis dissimilarity (*p* < 0.001, *F* = 11.72, *R*^2^ = 0.20, PERMANOVA) (Fig 4C). It is therefore possible that the metabolic pathways of ecosystems were different between upstream and downstream. The *in situ* experiment suggested the possibility that the drastic environmental change caused by treated wastewater contamination activated bacterial growth, including *Limnohabitans*, *Fluviicola*, and *Pseudarcicella*, and made bacterial community assembly deterministic.

### Microbial community assembly became deterministic in environments that differed from original environments

**Fig 5:**
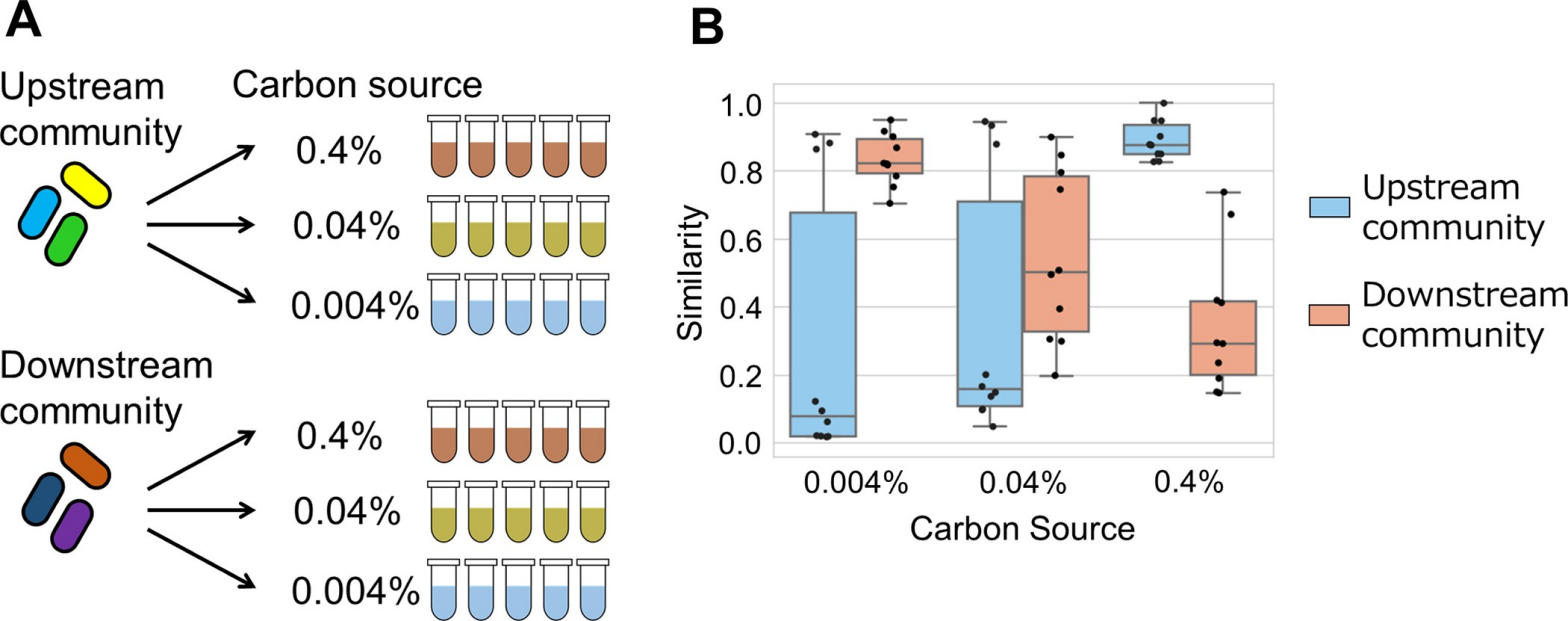
Stochasticity of bacterial community assembly in artificial conditions. **A.** Five replicates of the upstream bacterial community from site 2 and the downstream bacterial community from site 8 were cultivated in each glucose concentration: 0.4%, 0.04%, and 0.004%. **B.** Similarities of upstream/downstream bacterial compositions among replicates in each glucose condition are shown with box and jitter plot. Blue and orange boxes indicate upstream and downstream communities, respectively.

In the river experiment, in which upstream and downstream environments were quite different due to contamination of treated wastewater, downstream bacterial communities appeared to be more deterministic than upstream communities, but the influence of seasonal fluctuations may be included. Therefore, although the cultivation condition was quite different from the natural condition, the stochasticity of bacterial community assembly was examined by laboratory experiments that could control the environment. In this *ex situ* experiment, the environmental nutrient concentration changed for upstream and downstream bacterial communities to examine the causality between environment and stochasticity (Fig 5A). Since the results of community functional analysis suggested a difference between upstream and downstream bacterial communities (Fig 4C), and the fact that increased 16S rRNA gene amounts in the downstream community suggested a large biomass downstream (S7 Fig), downstream bacteria were presumed to be good at processing high-concentration organic matter and their metabolites. Thus, the carbon source concentration was varied in this *ex situ* experiment. In the upstream community, the community assembly was relatively deterministic in the high-concentration medium (similarity in 0.4% medium, 0.90 ± 0.06 SD among replicates) compared to that in the low concentration medium (0.004% medium, 0.31 ± 0.40 SD) (*p* = 0.009, *U* = 15, Mann-Whitney *U* two-sided test), and in the downstream community, these results were reversed (0.4% medium, 0.54 ± 0.21 SD; 0.004% medium, 0.83 ± 0.08 SD) (*p* = 0.005, *U* = 88, Mann-Whitney *U* two-sided test) (Fig 5B). The cell density of both upstream and downstream communities in 0.004% glucose medium was close to the limit of the device measurement (S12 Fig). In the medium containing 0.4% glucose, the genus *Pseudomonas* was dominant in all test tubes of upstream community (S13 Fig), while in 0.004% glucose, the genera *Aeromonas* and *Pantoea*, members of the family Enterobacteriaceae, and the phyla Patescibacteria were stochastically dominant. In the downstream community, the genera *Aeromonas* and *Klebsiella*, and a member of the family Enterobacteriaceae were mutually exclusively dominant in 0.4% glucose, while *Aeromonas* was dominant in all 0.004% glucose test tubes.

## Discussion

We investigated the possible factors controlling stochasticity of microbial community assembly with an *in silico* experiment that enabled the regulation of simple virtual microbial communities for comprehensive understanding, an *in situ* experiment observing original bacterial communities at various sites, and an *ex situ* experiment comparing bacterial communities assembled in artificial environments.

The *in silico* experiment showed that the assembly of bacterial communities was deterministic in a drastically changed environment with only a limited number of specialized microbial species. Stochastic assembly among replicates was confirmed with a mathematical model by adding the concept of essential bacteria, even if the nutrient intake trait differed among microbial species and was the same between replicates. Essential bacteria are required for the growth of each species. It has been estimated that only 1% of all archaea and bacteria can be cultivated *in vitro* [18–20], and the existence of essential bacteria has been mentioned as one of the reasons [21]. The exchange of essential metabolites and removal of inhibiting factors among bacteria have been examined *in vitro*, and have enabled the culturing of uncultured bacteria [22–24]. The exchange of metabolites, such as amino acids and sugars, in natural communities has also been systematically surveyed by using a metabolic modeling approach with over 800 microbial communities [25]. Although the *in silico* experiment assumed that traits differed among microbial species, neutral models assume identical traits for all species, and suggest that the limited species richness of the source metacommunity made the local community assembly more stochastic [26]. When considering only dispersion from a source community to a local community, the species richness of the source community will affect stochasticity of which species dominates.

The *in situ* experiment suggested that bacterial community assembly becomes deterministic with a large change in the river environment, due to the contamination of the treated wastewater between the natural upstream area and the urban downstream area. In the *ex situ* experiment with glucose as the only carbon source, the bacterial community from the relatively nutrient poor upstream area assembled deterministically with a high glucose concentration, and the bacterial community from the relatively nutrient rich downstream area assembled stochastically with a low glucose concentration.

A common finding in the *in silico* and *in situ* experiments was that the assembly of bacterial communities became deterministic when in a different environment. This phenomenon was also observed in other river communities. When two distinct upstream sites in the Chicago metropolitan region were compared, one of which was a highly urbanized river receiving effluent from a large wastewater treatment plant and the other was a suburban river receiving effluent from a much smaller wastewater treatment plant, it was discovered that they differed significantly in chemical characteristics and in the composition of their sediment bacterial communities [27]. However, downstream sites contaminated by treated wastewater became almost indistinguishable, where inorganic nutrients significantly increased, and biodiversity of sediment bacterial communities significantly decreased. In Brazil, two rivers also showed a decrease in biodiversity after being contaminated. The waters of the Sinos River, polluted downstream of Brazilian industrial centers, had a decreased Chao1 diversity for the microbial community, but further downstream (around 80 km from the pollution), the diversity recovered [28]. In our study, in the Tama River, we did not collect samples more than 25 km downstream from the treated wastewater contamination, therefore, the microbial community might have not yet adapted to the environmental change. The Chao1 diversity of water microbial community in the Jaboatão River in Brazil also decreased after being exposed to untreated wastewater [29]. The diversity did not recover further downstream, which may be because the most downstream samples were collected 20 km from the source of the pollution. On the other hand, the recovery of the sediment microbial community diversity at the most downstream site indicates that the microbial community adapts to the wastewater environmental pollution, because the sediment microbial community is less susceptible to river flow.

The phenomenon that environmental change results in a deterministic community assembly was observed not only in river communities, but also in communities of the groundwater-surface water mixing zone (the hyporheic zone) and gut. The hyporheic zone is dominated by river water microorganisms and low organic carbon concentrations at a high stage, and ground water microorganisms and high organic carbon concentration at a low stage; both communities are governed by a stochastic ecological process [30]. A rise in the river stage caused mixing of the groundwater and river water in the hyporheic zone and resulted in heterotrophic respiration. As a result, microbial taxa with particular ecological traits were selected and the community was governed by deterministic ecological processes. For human gut microbiota, a plant-based diet did not cause a change in the microbiota when compared with a standard diet, but the animal-based diet reproducibly caused a change in one day when compared with a standard diet [31]. The animal-based diet increased bile-tolerant microorganisms (the genera *Alistipes*, *Bilophila*, and *Bacteroides*) and decreased those that metabolize dietary plant polysaccharides (the genera *Roseburia*, *Eubacterium rectale*, and *Ruminococcus bromii* from the phylum Firmicutes). A meta-analysis of 27 dietary studies including 1,101 samples from rodents and humans also showed that a high-fat diet reproducibly changes the gut microbiota [32]. In particular, *Lactococcus* species increased with a high-fat diet and became one of the dominant bacteria. The number of bacterial species that adapt to a high-fat diet may be limited in the gut, which may make the assembly of bacterial communities deterministic. In support of this hypothesis, when microbiota in feces of omnivorous and herbivorous cattle were compared, the variation in characteristics between PC1 (24%) and PC2 (15%) in PCA was larger in herbivores, suggesting that gut microbiota adapted to herbivores become deterministic with an omnivorous diet [33].

The *in silico* and *ex situ* experiments showed the convergence of microbial component patterns. Limited species richness was the key to converge microbial community assembly in the *in silico* experiment. The microbial composition was called a pattern if, when the initial amounts of microorganisms randomly chosen with the same parameter set and the calculation was performed 10 times *in silico*, a similar microbial community appeared more than once (Renkonen similarity ≥ 0.9). The probability of pattern occurrence was 83.0% with species richness due to the supply of resource concentration adapted by the microbial communities in our *ex situ* experiment. However, the probability of pattern occurrence was 97.5% when there was a poor species richness due to disagreement between the community adapted and supplied concentration.

As patterns were more likely to occur in an environment where microbial community assemblies were reproducible among replicates *in silico*, emergent patterns of microbial components were also observed *ex situ*. In the *ex situ* experiment with five replicates, three patterns of microbial community appeared: (i) *Pseudomonas* dominant, (ii) *Aeromonas* and *Acinetobacter* dominant, and (iii) *Klebsiella* and *Acinetobacter* dominant (S4 Table). When cultivating the upstream-river microbial community, pattern (i) appeared all five times with a 0.4% glucose medium, pattern (ii) appeared three times and pattern (iii) appeared one time with a 0.04% glucose medium, and pattern (ii) appeared three times with a 0.004% glucose medium. When cultivating the downstream-river microbial community, pattern (ii) appeared two times and pattern (iii) appeared two times with a 0.4% glucose medium, pattern (ii) appeared three times and pattern (iii) appeared two times with a 0.04% glucose medium, and pattern (ii) appeared all five times with 0.004% glucose medium. The dominant bacteria *Pseudomonas*, *Aeromonas*, *Acinetobacter*, and *Klebsiella* spp. can utilize a wide range of carbon sources [34–37]. *Aeromonas* and *Acinetobacter* dominant in pattern (ii) were observed to be dominant in inflow water to wastewater treatment plants and were adapted to the sewer infrastructure environment [38]. As *Aeromonas* and *Acinetobacter* dominated even in our 0.004% glucose medium, regarded as a low carbon concentration in our experiment, the *ex situ* experiment was limited by the fact that various organic and inorganic substances were different between the natural and cultivation environments. However, even under this limitation, patterns were more probable when the upstream microbial community was in high carbon concentration cultures, and the downstream microbial community was in low carbon concentration cultures. Similarly, a previous study, in which microbial communities were cultivated in glucose limited M9 medium (70 mM glucose concentration close to our 0.4% ≈ 20 mM glucose medium), observed functional self-organization by cross-feeding between a functional guild metabolizing glucose and secreting acetate, lactate, and succinate, as primary byproducts, and another guild metabolizing the same secretions [39]. The former guild possessed the phylogenetic families Pseudomonadaceae and Moraxellaceae, and the later guild possessed Enterobacteriaceae and Aeromonadaceae. As *Aeromonas* dominating in pattern (ii) and *Klebsiella* dominating in pattern (iii) belong to Aeromonadaceae, and *Acinetobacter* dominating in both patterns belongs to Moraxellaceae, the same functional self-organization would occur in our *ex situ* experiment. Although *Pseudomonas* belongs to Pseudomonadaceae, it was speculated that *Pseudomonas* was also superior in glucose utilization in our experiment. The reason why microbial community assembly became deterministic in a drastically changed environment may be that the number of microbial species adapted to the new environment and the possible patterns of microbial composition were limited.

Herein, we propose that the initial species richness make the community assembly stochastic, as demonstrated by *in silico* experiments. In addition, initial species evenness can influence the community assembly, which previously reported that initial community evenness can preserve the functional stability of a community [40]. The stability of the community denitrification upon environmental stress can be strongly influenced by the initial evenness of the microbial community in which many microorganisms have similar functions as alternatives. This functional stability and stochastic assembly influenced by species richness herein reported may be closely related.

In the present study, the *in situ* experiment revealed the phenomenon that drastic environmental change caused the deterministic assembly of the microbial community, which was further confirmed by the *in silico* experiment. As the mathematical model interpretation of the *in situ* experiment results, the *in silico* experiment proposed a mechanism of the phenomenon in which the number of microbial species adapted to the environment after drastic change is limited and makes the community assembly deterministic. Nevertheless, it remains unknown how many microbial species adapted to the environment after drastic change in *in situ* and *ex situ* experiments, since the functions of the microorganisms were not measured in this study. Therefore, it is still necessary to measure transcripts and metabolites for analyzing the functional aspects of this phenomenon. For the microbial growth in *in silico* experiment, not only resource availability but also resource quality should be considered in the mathematical model. It has been reported that the quality of resources, such as what metabolic by-products are produced from a resource and the combination of resources, influences the assembly of microbial communities [39, 41]. For the mathematical model, the phenomenon was reproduced with many parameter sets in this study, but it is also important to identify the parameters of the mathematical model from *in situ* data by larger field sampling in the future.

In summary, drastic environmental change caused the deterministic assembly of the microbial community *in silico*, *in situ*, and *ex situ*. This rule appears to be used in microbial communities in the river, the gut, and the hyporheic zone between groundwater and surface water, but it is also necessary to show whether the bacterial communities in other environments are also governed by this rule. This proposed rule of microbial community assembly will help us to understand the mechanism of stochastic processes and control the systems where microorganisms are utilized.

## Supporting information

S1 Fig Microbial communities adapted to supplied resource concentration. **A.** The community adapted to a high resource concentration consisted of a large number of species adapted to high supplied resource concentration and a small number of species adapted to low supplied resource concentration. Arrows from resources to microbial species indicate resource uptake and opposite arrows indicate secretion of metabolic byproducts. All resources are associated with specialized microbial species, as indicated by thick arrows. Blue and orange thick arrows indicate supplied resource uptake of high and low resource concentration adapted microbial species, respectively. Gray thick arrows indicate other resource uptake of microbial species specialized for the resource. Gray thin arrows indicate resource uptake of microbial species non-specialized for the resource and secretion of metabolic byproducts. **B.** The community adapted to a low resource concentration have the opposite composition.
(TIF)

S2 FigSpecialization for a supplied resource concentration and *in silico* experiment for some conditions of a supply resource concentration.**A.** Microbial species specialized for a supplied resource are activated by the specific supplied resource concentration as $G_{i\alpha }=4\frac{I_{\alpha }}{L_{i}+I_{\alpha }}\frac{L_{i}}{L_{i}+I_{\alpha }}$, in which *I*_*α*_ is the supply concentration of the resource *α* in the medium and *L*_*i*_ represent a value similar to the Michaelis constant. Blue line indicates metabolic activity of microbial species adapted to low concentration *I*_*α*_ = 1, where *L*_*i*_ = 1. Orange line indicates metabolic activity of microbial species adapted to high concentration *I*_*α*_ = 100, where *L*_*i*_ = 100. **B.** As the supplied resource concentration, 0.5, 1, 10, 100, and 200, as indicated by dashed lines in the panel **A**, were examined for communities adapted to high and low resource concentration, respectively. Orange boxes indicate the similarity of community adapted to high resource concentration consisted of 45 and five species adapted to a high and low resource concentration, respectively, for a supplied resource, and 10 species specialized for each of the other resources. Blue boxes indicate that of community adapted to low resource concentration.(TIF)

S3 Fig Flow for numerical calculation and Renkonen similarity in *in silico* experiments. After setting the parameters for species-specific resource consuming rate *C*_*iα*,_ stoichiometric matrix of resources *D*^*i*^_*αβ*_, and target as essential symbionts, each numerical calculation was performed 10 times by changing the initial biomass, which was randomly selected between 0.01 and 1. Renkonen similarity was calculated for each of the 10 replicates using the final microbial compositions. The calculations were repeated 100 times.
(TIF)

S4 Fig Sampling sites in the Tama River. Treated wastewater from several wastewater treatment plants contaminates the river between sites 4 and 6. The map is based on the Digital Map published by Geospatial Information Authority of Japan (2021).
(TIF)

S5 Fig The result of ion chromatography in the Tama River. Concentrations of NO_2_^-^, PO_4_^3-^, SO_4_^2-^, Na^+^, K^+^, Mg^2+^, and Ca^2+^ at eight sites from August 31, 2018 to July 21, 2019.
(TIF)

S6 Fig The result of dissolved organic carbon in the Tama River. Concentrations of dissolved organic carbon at eight sites from August 31, 2018 to July 21, 2019.
(TIF)

S7 Fig 16S rDNA amounts quantified by real time-PCR. 16S rDNA amounts per mL at eight sites from August 31, 2018 to July 21, 2019. The black line indicates the median among six time points.
(TIF)

S8 Fig Observed operational taxonomic units (OTUs) at sampling sites before (blue) and after (orange) treated wastewater contamination. 16S rRNA analysis data from eight sites between August 31, 2018 to July 21, 2019 was used for the count of OTUs.
(TIF)

S9 Fig Bacterial composition at each taxonomic level in the Tama River at six time points from August 31, 2018 to July 21, 2019. The Tama River samples collected from eight sites at each time point. The most abundant eight taxa at each taxonomic level are listed.
(TIF)

S10 Fig Relative abundances of *Limnohabitans*, *Fluviicola*, and *Pseudarcicella* along the Tama River. Solid lines indicate means of relative abundance and shadows represent the respective 95% confidence intervals. Blue, orange, and green lines correspond to *Limnohabitans*, *Fluviicola*, and *Pseudarcicella*, respectively.
(TIF)

S11 Fig Principal coordinate analysis (PCoA) biplot based on Bray-Curtis dissimilarity of microbial composition in the Tama River. For the biplot, environmental factors (temperature, pH, dissolved organic carbon [DOC], NO^2-^, PO_4_^3-^, SO_4_^2-^, Na^+^, K^+^, Mg^2+^, Ca^2+^) were used and shown (*p* < 0.05). The numbers indicate the site in the Tama River.
(TIF)

S12 Fig The optical density of both upstream and downstream communities in 0.4%, 0.04%, and 0.004% glucose measured every 2 days. Each graph includes five lines indicating five replicates.
(TIF)

S13 Fig Upstream and downstream bacterial composition at each taxonomic level cultivated in 0.4%, 0.04%, and 0.004% glucose. Five replicates for each condition. Up and Down indicate bacterial composition sourced from upstream and downstream in the Tama River on September 7, 2019, respectively. The most abundant eight taxa at each taxonomic level are listed.
(TIF)

S1 Table Variables and parameters included in the mathematical model. (XLSX)

S2 Table Water temperature [°C] in the Tama River at six time points. Temperature at eight sites from August 31, 2018 to July 21, 2019.
(XLSX)

S3 Table pH in the Tama River at six time points. pH at eight sites from August 31, 2018 to July 21, 2019.
(XLSX)

S4 Table Community patterns in the *ex situ* experiment with five replicates. The number of emergent community patterns is shown. Three patterns of microbial community appeared: (i) *Pseudomonas* dominant, (ii) *Aeromonas* and *Acinetobacter* dominant, and (iii) *Klebsiella* and *Acinetobacter* dominant.
(XLSX)

S1 Dataset The amplicon sequence variant table of *in situ* experiment. The number of reads of each amplicon sequence variant for *in situ* experiment is shown.
(CSV)

S2 Dataset The amplicon sequence variant table of *ex situ* experiment. The number of reads of each amplicon sequence variant for *ex situ* experiment is shown.
(CSV)
