# Supplementary figures and images for: Determinism of microbial community assembly by drastic environmental change
Source: PLoS One. 2021 Dec 2;16(12):e0260591. doi: 10.1371/journal.pone.0260591 (PMC8638896; doi:10.1371/journal.pone.0260591)

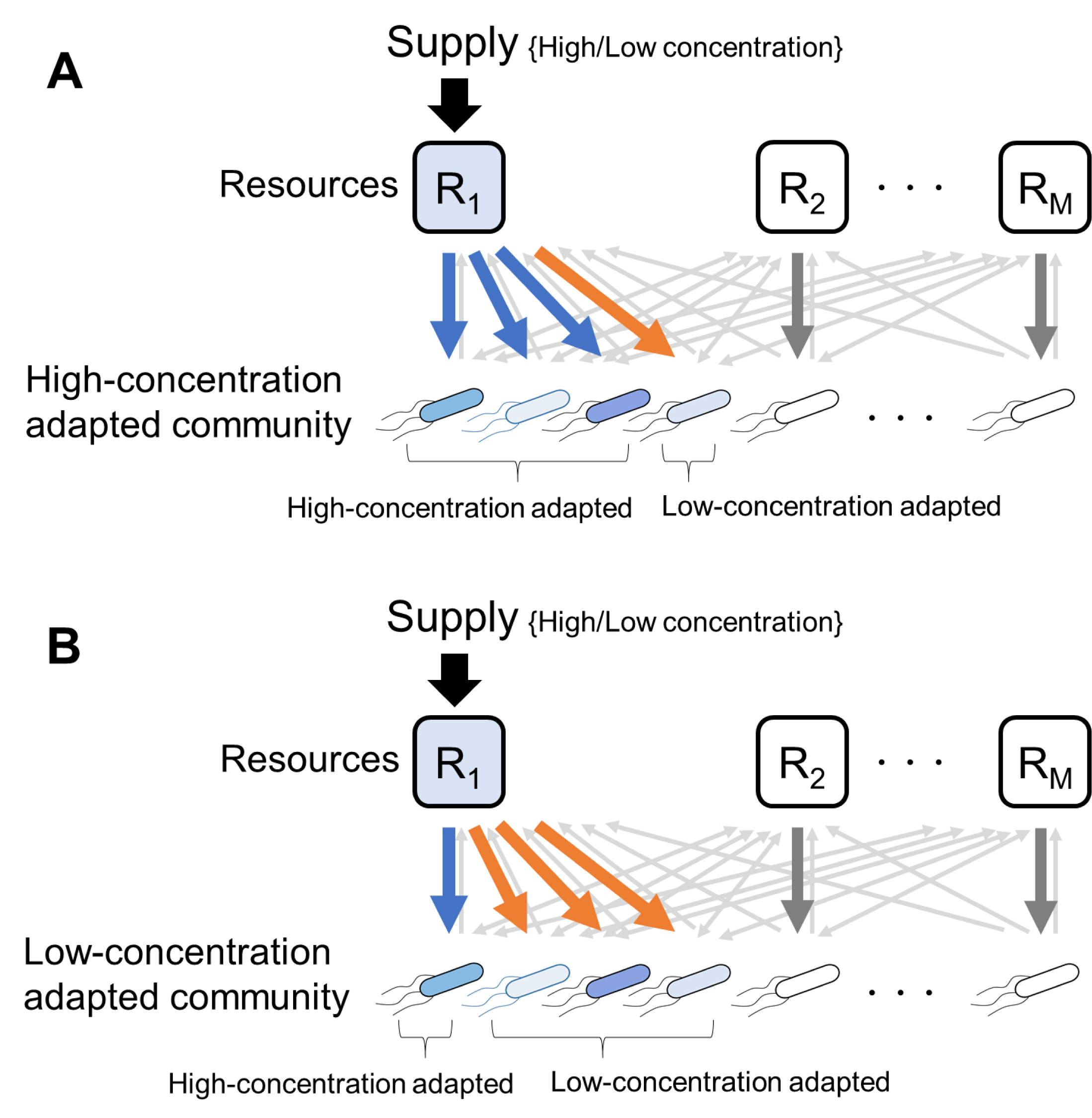

Supplement: S1 Fig — A. The community adapted to a high resource concentration consisted of a large number of species adapted to high supplied resource concentration and a small number of species adapted to low supplied resource concentration. Arrows from resources to microbial species indicate resource uptake and opposite arrows indicate secretion of metabolic byproducts. All resources are associated with specialized microbial species, as indicated by thick arrows. Blue and orange thick arrows indicate supplied resource uptake of high and low resource concentration adapted microbial species, respectively. Gray thick arrows indicate other resource uptake of microbial species specialized for the resource. Gray thin arrows indicate resource uptake of microbial species non-specialized for the resource and secretion of metabolic byproducts. B. The community adapted to a low resource concentration have the opposite composition. (TIF) [file pone.0260591.s001.tif]

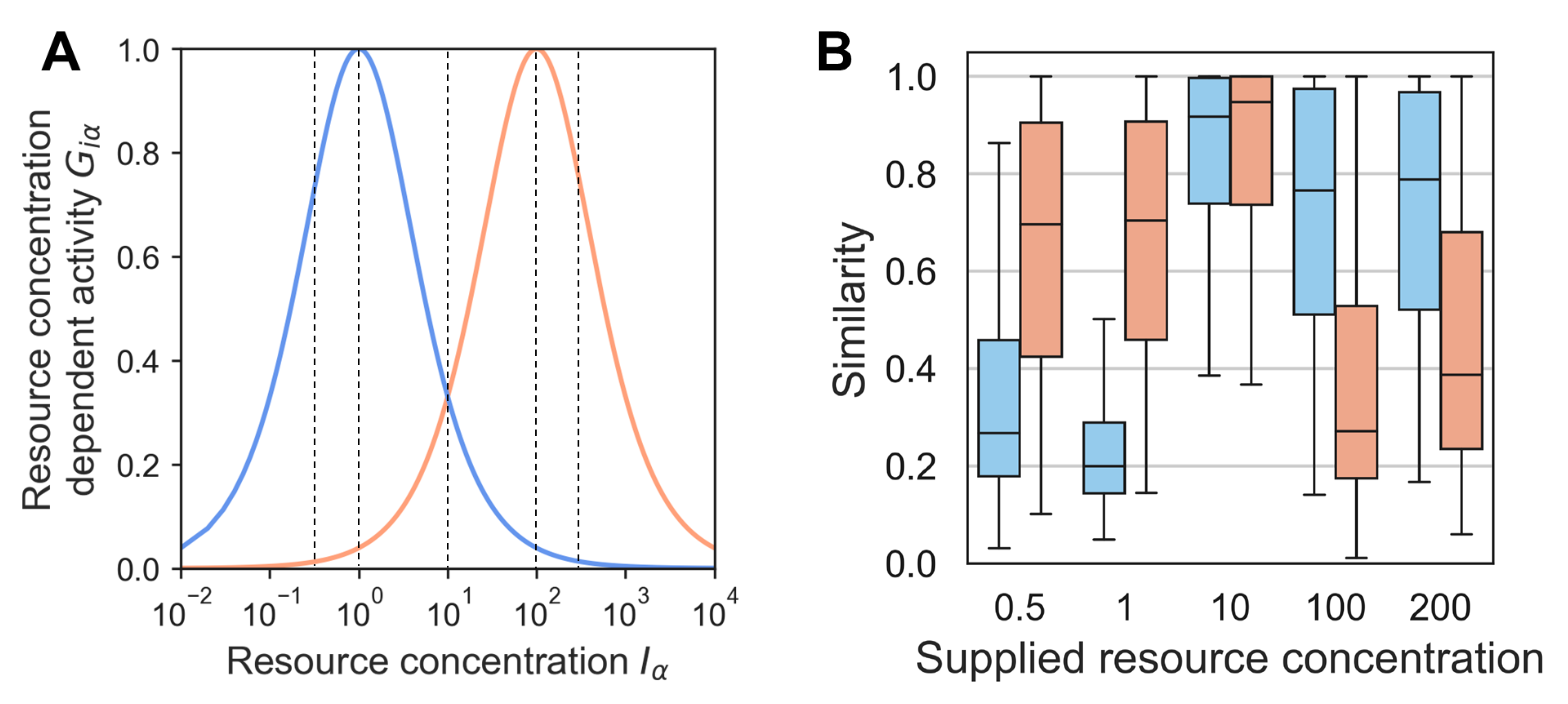

Supplement: S2 Fig — A. Microbial species specialized for a supplied resource are activated by the specific supplied resource concentration as Giα=4IαLi+IαLiLi+Iα, in which Iα is the supply concentration of the resource α in the medium and Li represent a value similar to the Michaelis constant. Blue line indicates metabolic activity of microbial species adapted to low concentration Iα = 1, where Li = 1. Orange line indicates metabolic activity of microbial species adapted to high concentration Iα = 100, where Li = 100. B. As the supplied resource concentration, 0.5, 1, 10, 100, and 200, as indicated by dashed lines in the panel A, were examined for communities adapted to high and low resource concentration, respectively. Orange boxes indicate the similarity of community adapted to high resource concentration consisted of 45 and five species adapted to a high and low resource concentration, respectively, for a supplied resource, and 10 species specialized for each of the other resources. Blue boxes indicate that of community adapted to low resource concentration. (TIF) [file pone.0260591.s002.tif]

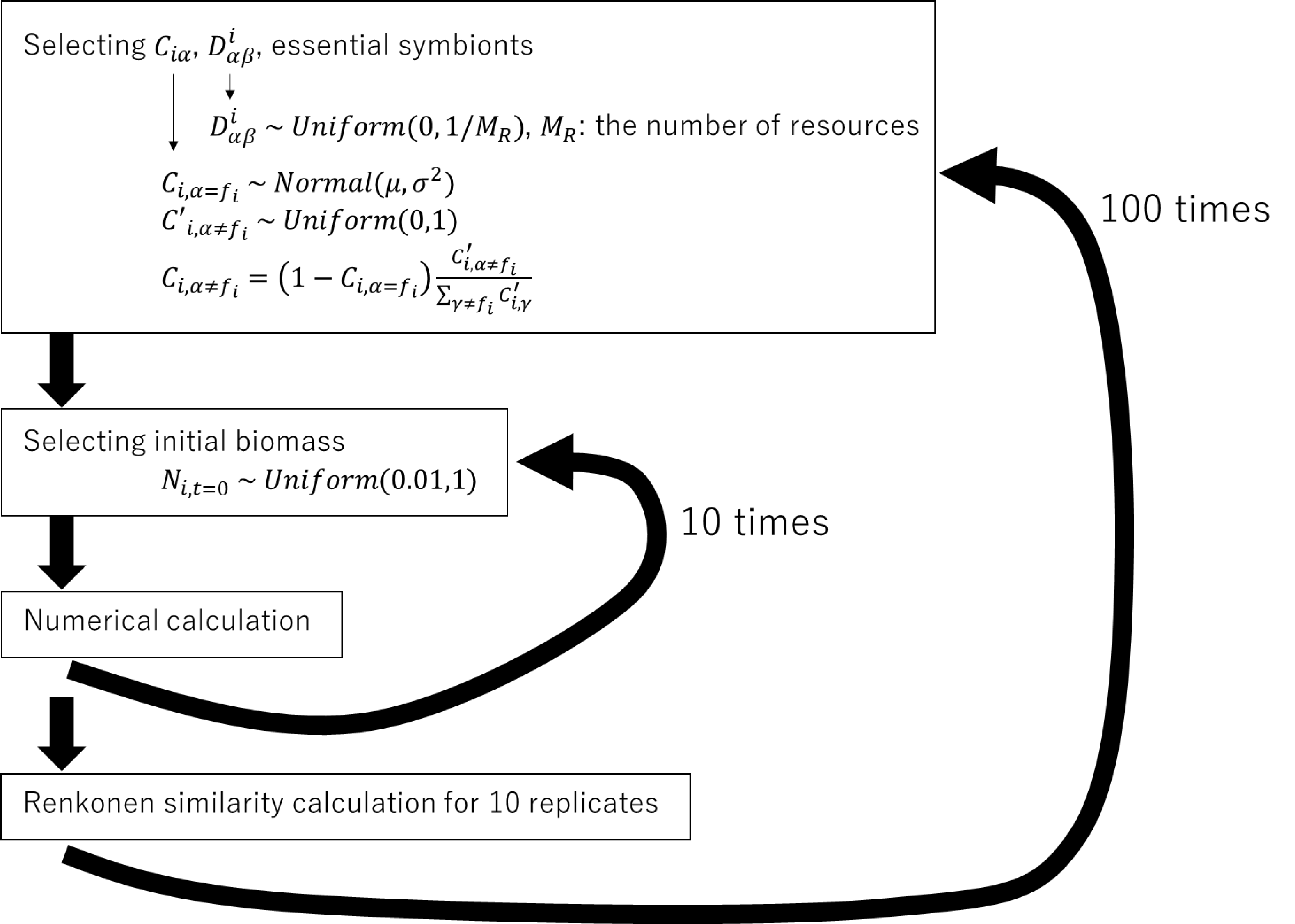

Supplement: S3 Fig — After setting the parameters for species-specific resource consuming rate Ciα, stoichiometric matrix of resources Diαβ, and target as essential symbionts, each numerical calculation was performed 10 times by changing the initial biomass, which was randomly selected between 0.01 and 1. Renkonen similarity was calculated for each of the 10 replicates using the final microbial compositions. The calculations were repeated 100 times. (TIF) [file pone.0260591.s003.tif]

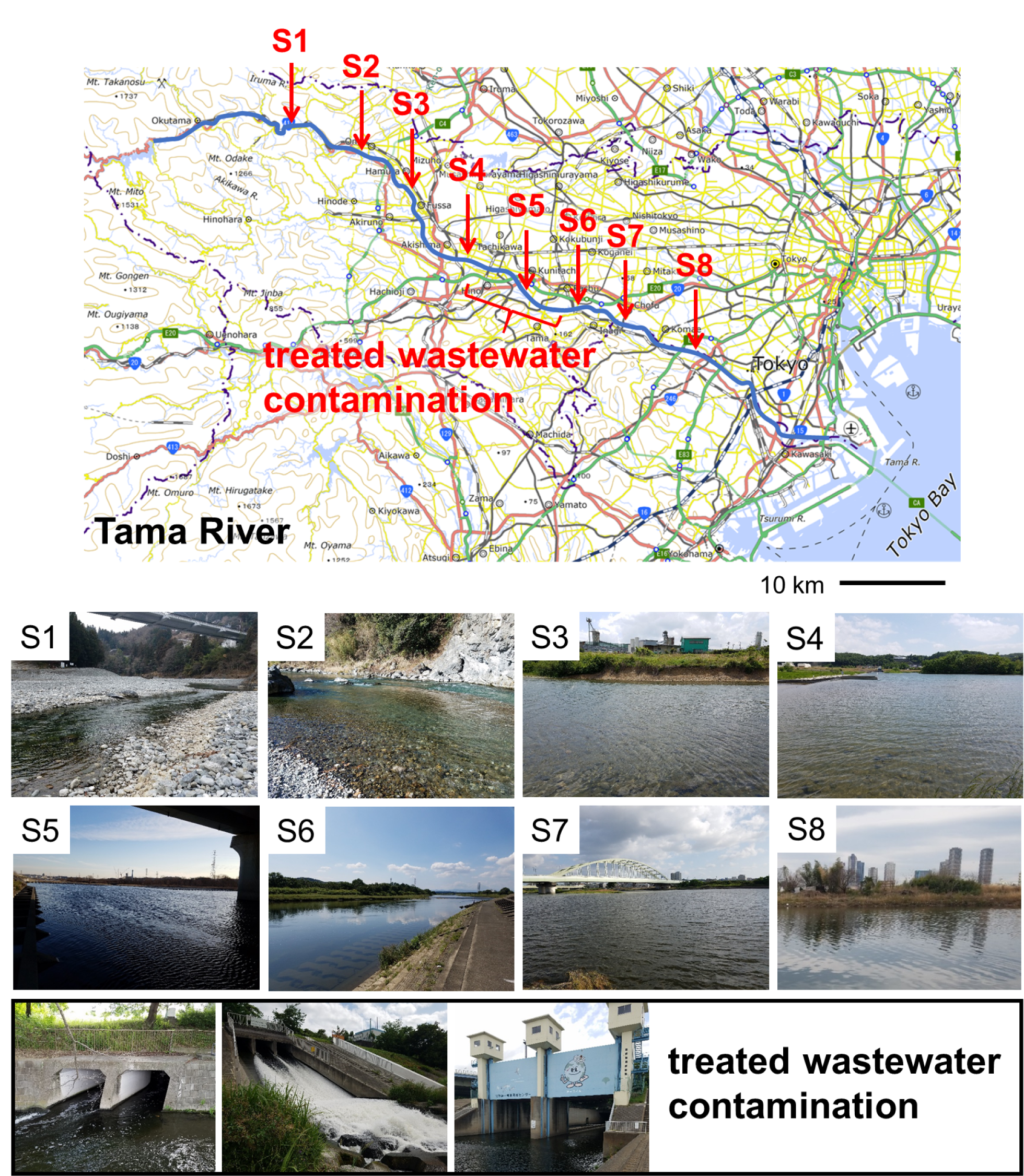

Supplement: S4 Fig — Treated wastewater from several wastewater treatment plants contaminates the river between sites 4 and 6. The map is based on the Digital Map published by Geospatial Information Authority of Japan (2021). (TIF) [file pone.0260591.s004.tif]

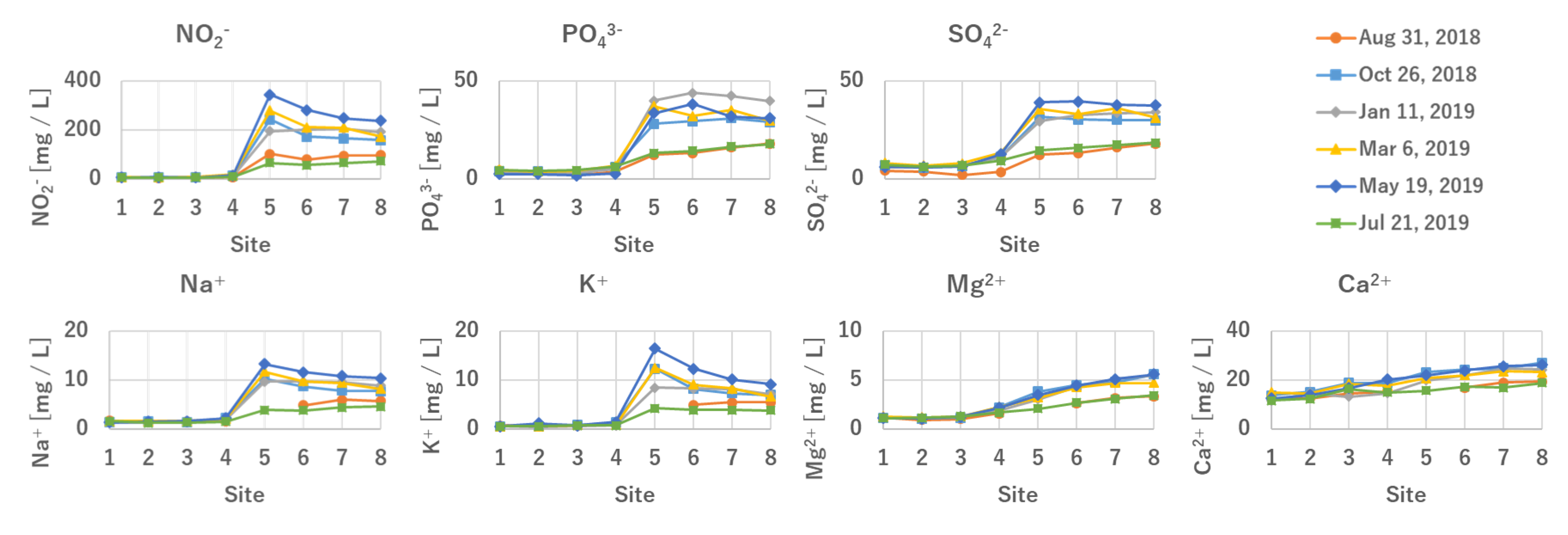

Supplement: S5 Fig — Concentrations of NO2-, PO43-, SO42-, Na+, K+, Mg2+, and Ca2+ at eight sites from August 31, 2018 to July 21, 2019. (TIF) [file pone.0260591.s005.tif]

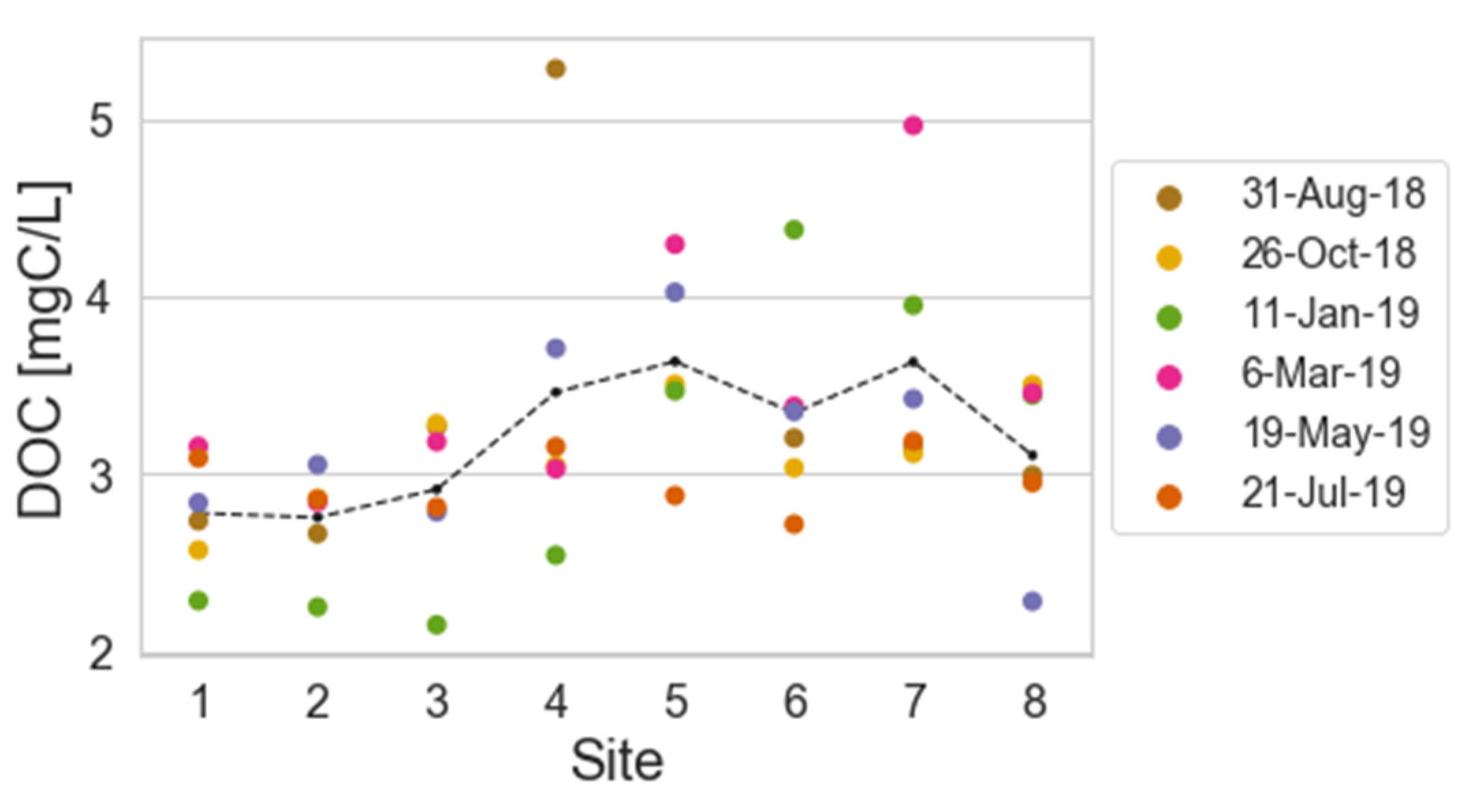

Supplement: S6 Fig — Concentrations of dissolved organic carbon at eight sites from August 31, 2018 to July 21, 2019. (TIF) [file pone.0260591.s006.tif]

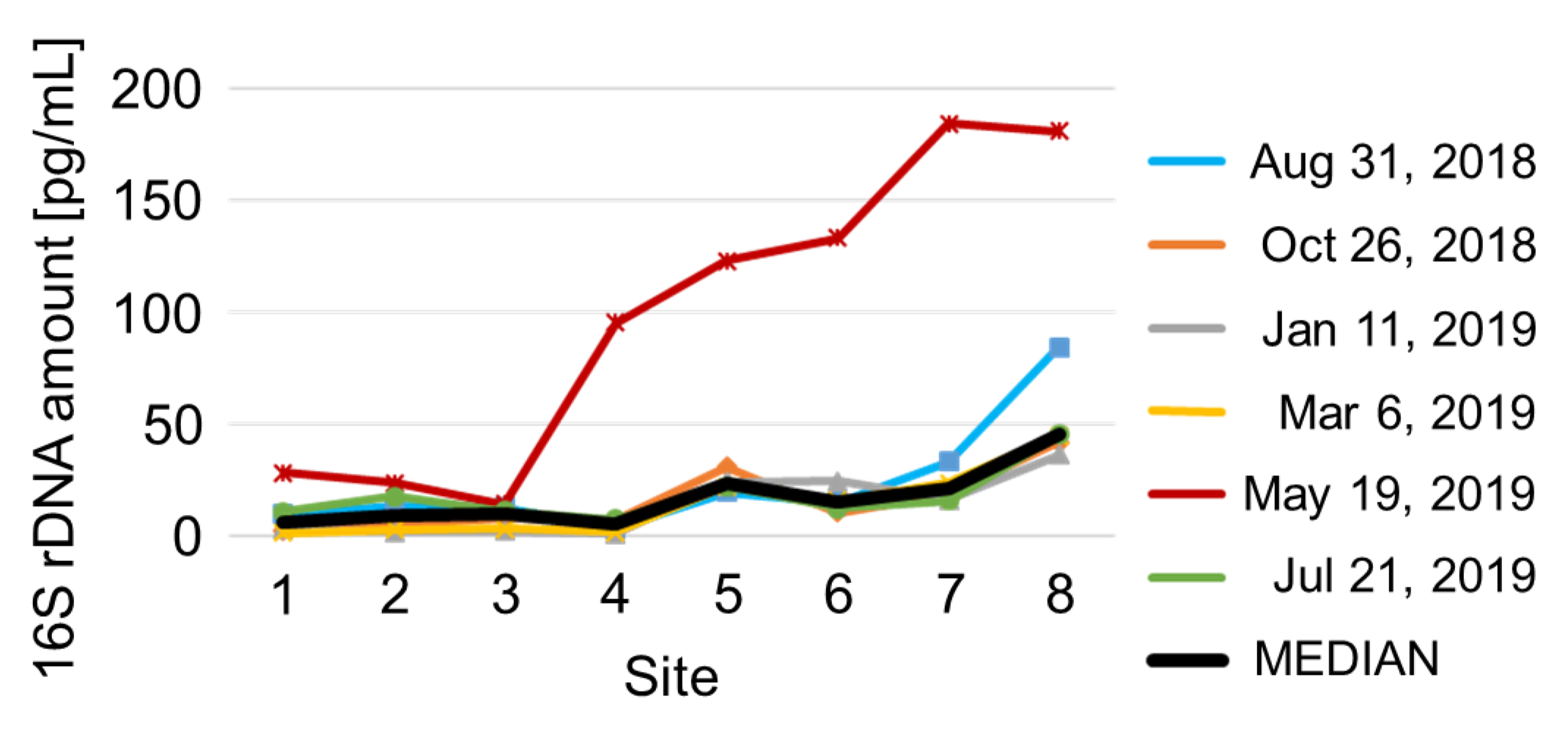

Supplement: S7 Fig — 16S rDNA amounts per mL at eight sites from August 31, 2018 to July 21, 2019. The black line indicates the median among six time points. (TIF) [file pone.0260591.s007.tif]

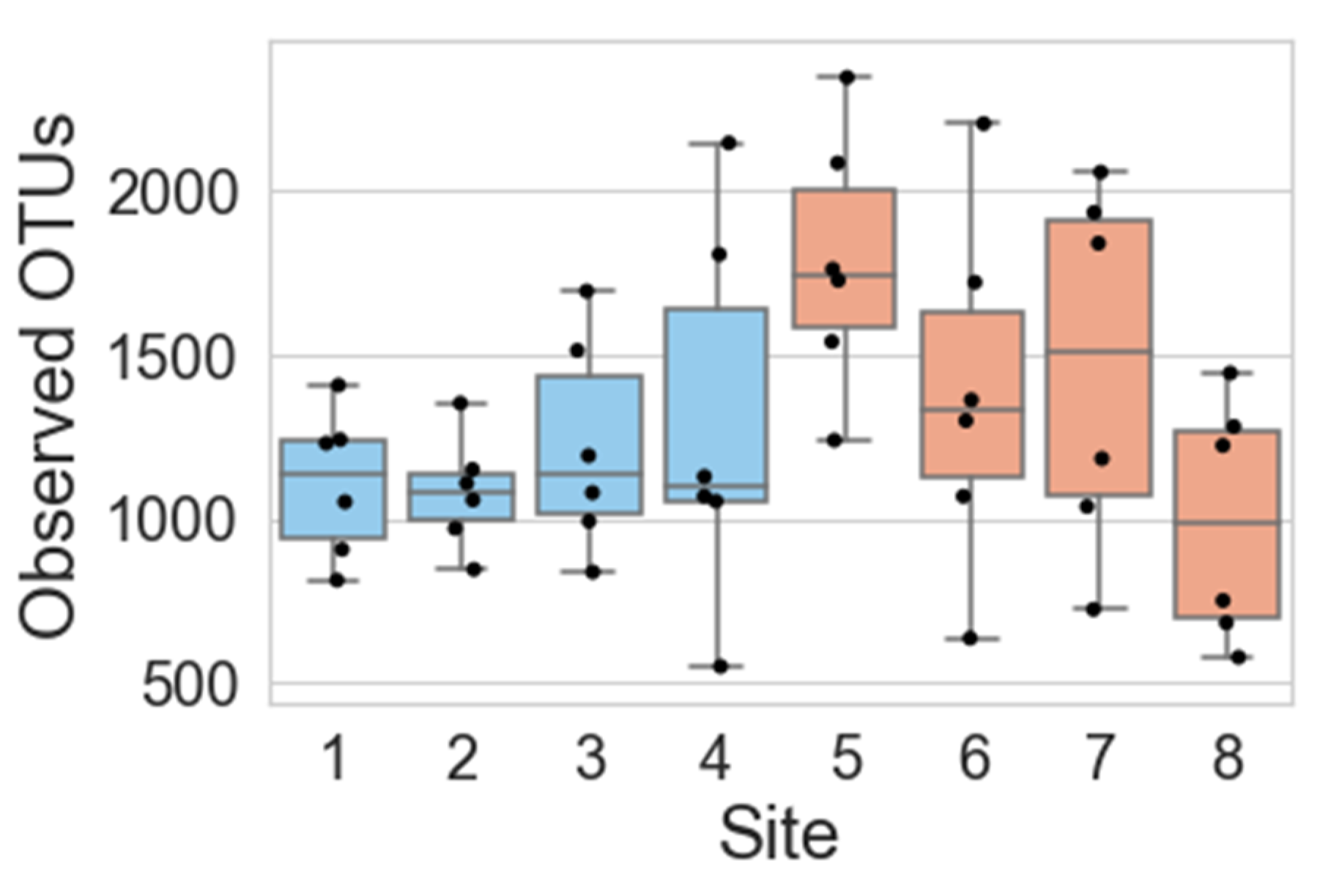

Supplement: S8 Fig — 16S rRNA analysis data from eight sites between August 31, 2018 to July 21, 2019 was used for the count of OTUs. (TIF) [file pone.0260591.s008.tif]

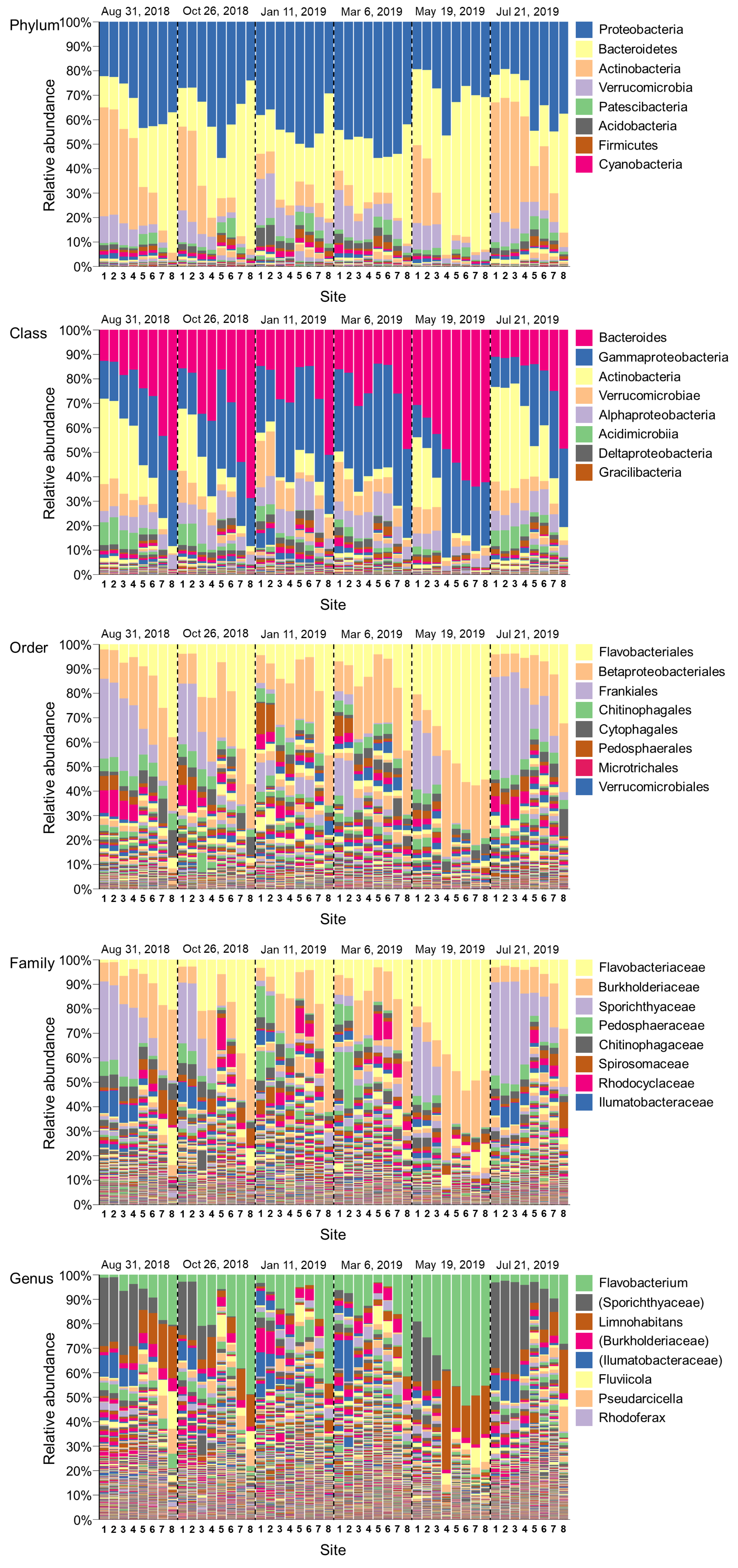

Supplement: S9 Fig — The Tama River samples collected from eight sites at each time point. The most abundant eight taxa at each taxonomic level are listed. (TIF) [file pone.0260591.s009.tif]

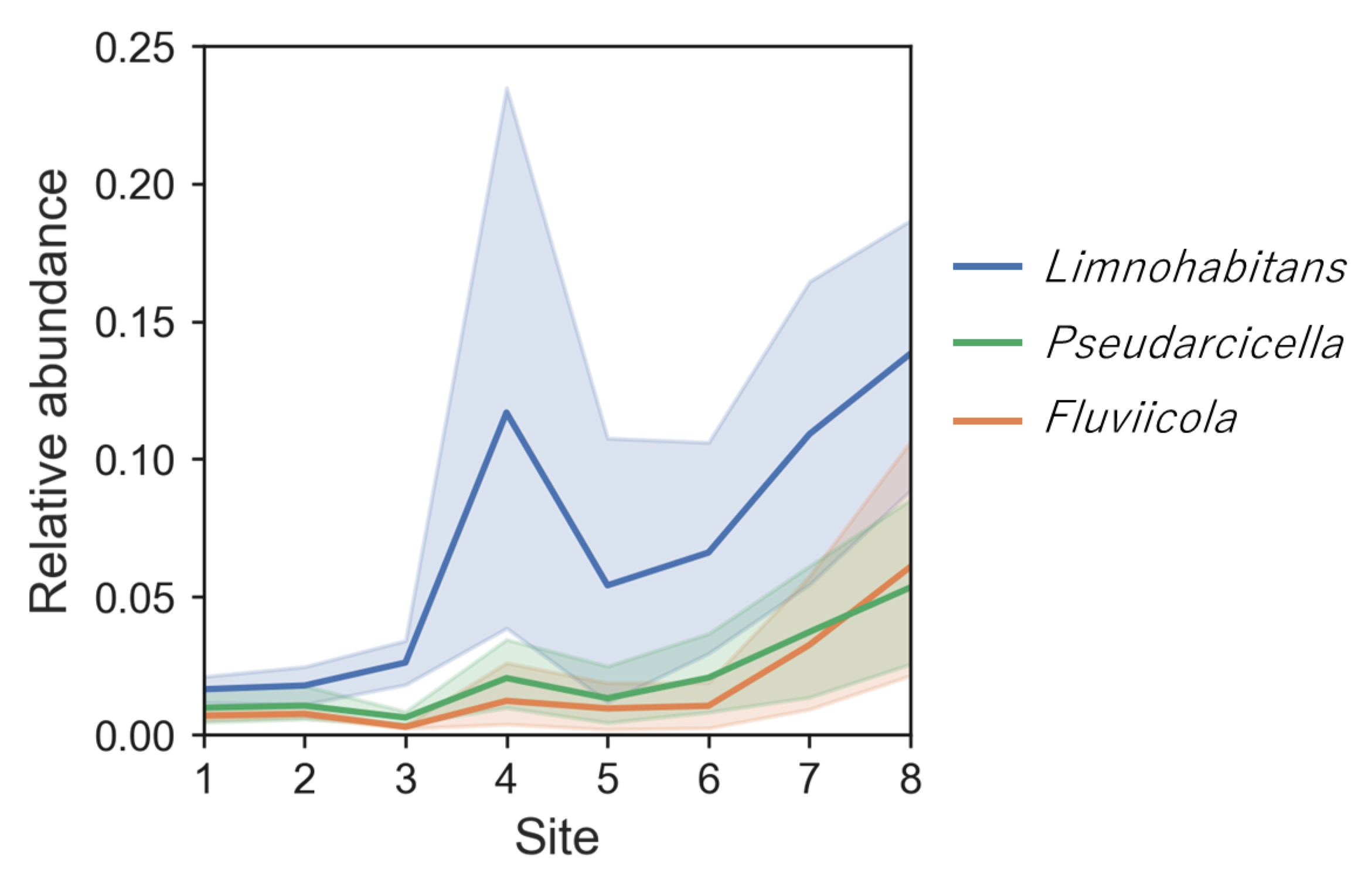

Supplement: S10 Fig — Solid lines indicate means of relative abundance and shadows represent the respective 95% confidence intervals. Blue, orange, and green lines correspond to Limnohabitans, Fluviicola, and Pseudarcicella, respectively. (TIF) [file pone.0260591.s010.tif]

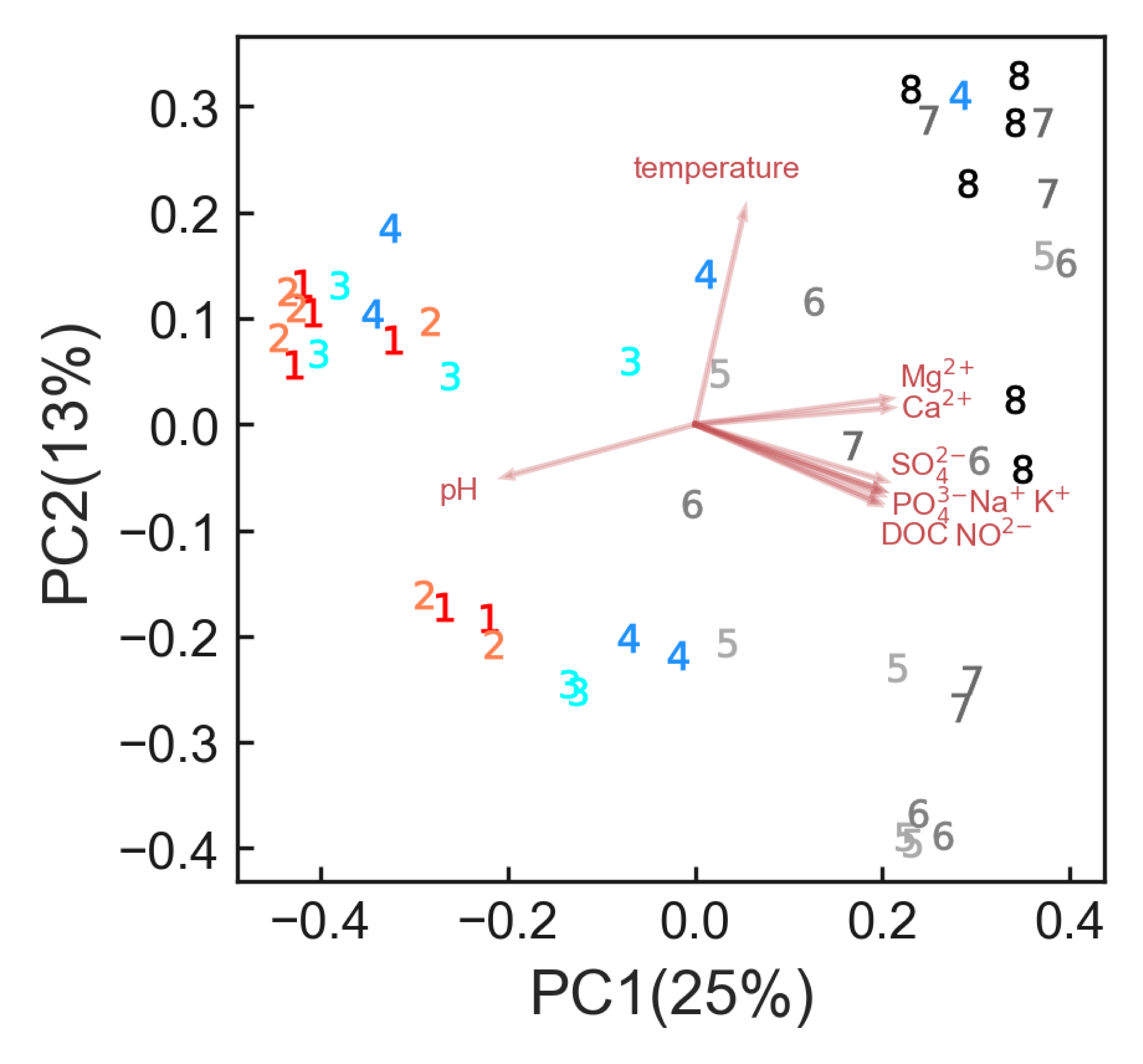

Supplement: S11 Fig — For the biplot, environmental factors (temperature, pH, dissolved organic carbon [DOC], NO2-, PO43-, SO42-, Na+, K+, Mg2+, Ca2+) were used and shown (p < 0.05). The numbers indicate the site in the Tama River. (TIF) [file pone.0260591.s011.tif]

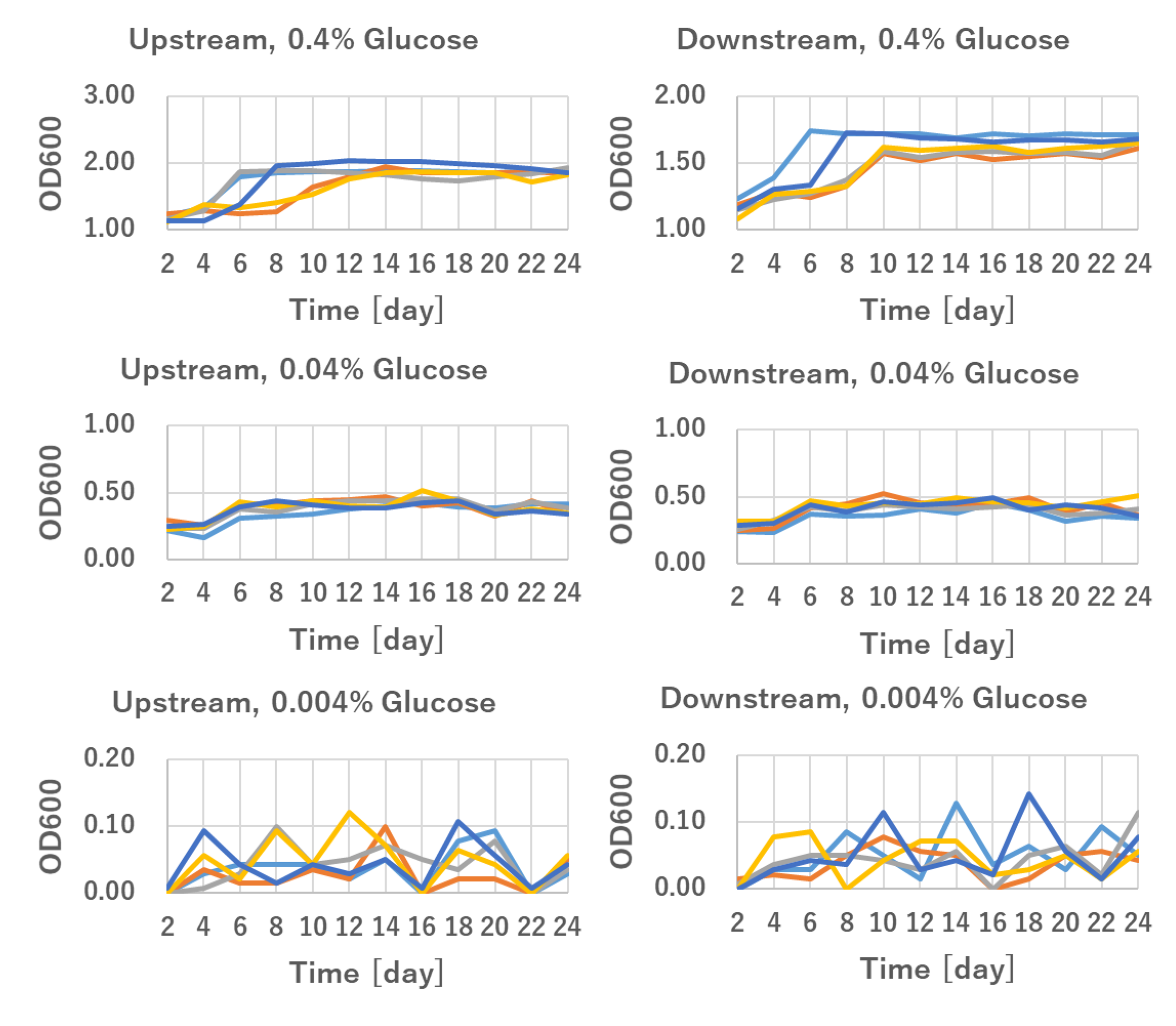

Supplement: S12 Fig — Each graph includes five lines indicating five replicates. (TIF) [file pone.0260591.s012.tif]

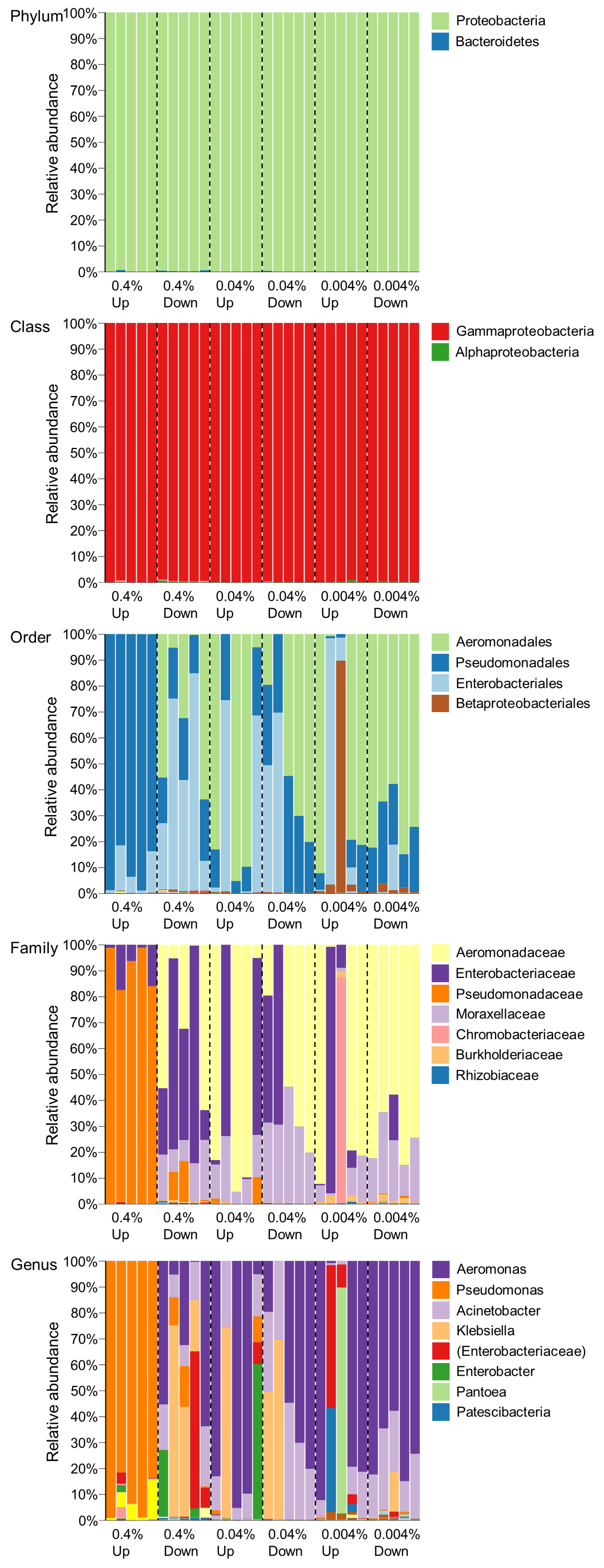

Supplement: S13 Fig — Five replicates for each condition. Up and Down indicate bacterial composition sourced from upstream and downstream in the Tama River on September 7, 2019, respectively. The most abundant eight taxa at each taxonomic level are listed. (TIF) [file pone.0260591.s013.tif]
